# Biocatalytic Cascade from Geraniol to Enantiopure (*R*)‑Citronellal Using an Aryl-Alcohol Oxidase and Ene Reductase

**DOI:** 10.1021/acs.orglett.6c02985

**Published:** 2026-09-09

**Authors:** Claudia Ferrer-Carbonell, Christian Ascaso-Alegre, Paula Cinca-Fernando, Patricia Ferreira, Juan Mangas-Sánchez, Caroline E. Paul

**Affiliations:** ‡ Biocatalysis Section, Department of Biotechnology, 2860 Delft University of Technology, Van der Maasweg 9, 2629 HZ Delft, Netherlands; § Departamento de Bioquímica y Biología Molecular y Celular, Facultad de Ciencias, 16765 Universidad de Zaragoza, 50009 Zaragoza, Spain; ∥ Instituto de Biocomputación y Física de Sistemas Complejos, BIFI (GBsC−CSIC Joint Unit), Universidad de Zaragoza, 50018 Zaragoza, Spain; ⊥ Department of Organic and Inorganic Chemistry, IUQEM, University of Oviedo, Julián Clavería 8, 33006 Oviedo, Spain

## Abstract

(*R*)-Citronellal, a key intermediate for (−)-menthol and other valuable fragrance compounds, is traditionally synthesized by unsustainable chemical methods, with low selectivity. Herein, we report a one-pot enzymatic cascade converting geraniol to enantiopure (*R*)-citronellal using *Streptomyces hiroshimensis* aryl-alcohol oxidase *Sh*AAO and ene reductase *Saccharomyces cerevisiae* OYE2_Y83V. A scale-up reaction at 100 mM geraniol produced 95 mg of (*R*)-citronellal with an enantiomeric excess of >99.8%, demonstrating a sustainable, preparative-scale, and highly selective route to chiral terpenoids.

## Graphical abstract

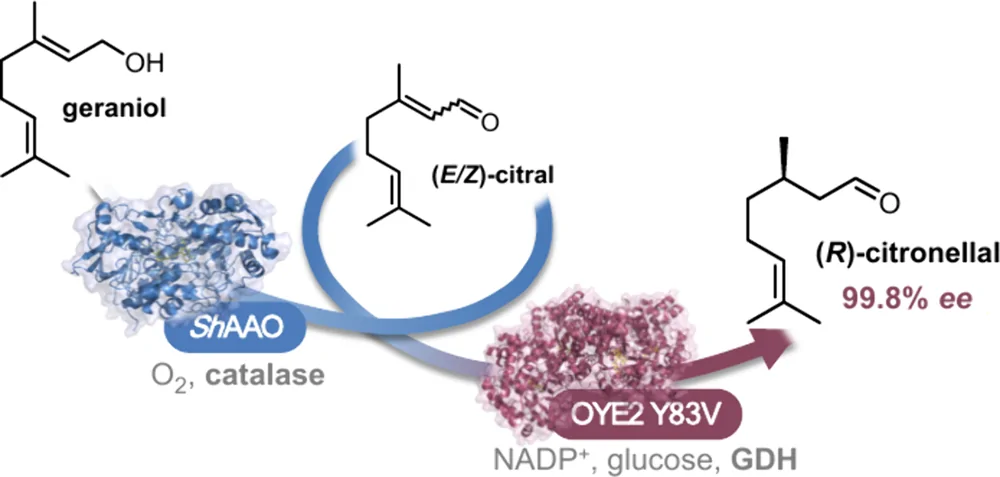

Chiral terpenoids constitute valuable synthetic targets due to their widespread applications as flavors, fragrances, agrochemicals, and pharmacologically active compounds.^1^ Among them, (*R*)-citronellal is a versatile chiral building block in organic synthesis, providing access to a wide variety of stereoselective transformations, including cyclizations, reductions, oxidations, and coupling reactions.^2^ Through these established routes, (*R*)-(+)-citronellal enables access to high-value molecules, such as l-menthol,^3,4^ caparratriene,^5^ and chalcogen-containing nitrone derivatives (Scheme 1A).^6^

**Scheme 1:**
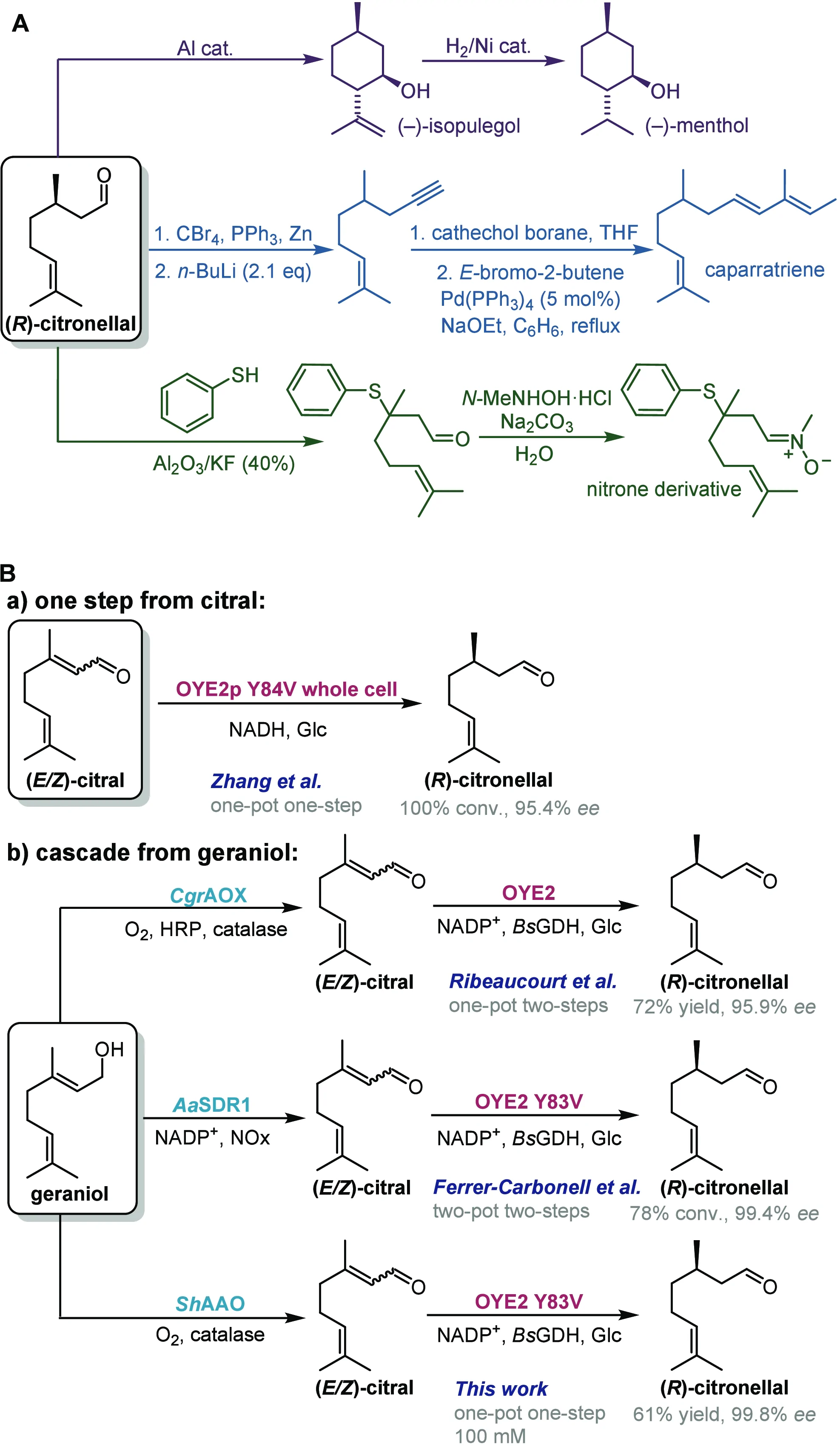
(A) Synthetic Routes from (*R*)-Citronellal to Valuable Derivatives, Including (−)-Menthol, Caparratriene, and Chalcogen-Containing Nitrone Derivatives, and (B) State of the Art in the Enzymatic Synthesis of (*R*)-Citronellal

In the fragrance industry, (*R*)-citronellal is a key chiral precursor in the large-scale production of (−)-menthol, one of the most highly consumed aroma compounds worldwide.^4,7,8^ To meet this high demand, the most well-known synthetic routes for (−)-menthol production either start with bio-based myrcene (Takasago), fossil-based *m*-cresol (Symrise), or versatile citral (BASF), a mixture of *E*/*Z* isomers geranial and neral.^4,9^ For instance, the commercial route of BASF relies on the catalytic hydrogenation of citral to citronellal, followed by cyclization to isopulegol and subsequent hydrogenation to menthol.^4,8^ However, this process generally yields 87% enantiomeric excess (*ee*) (*R*)-citronellal, which requires downstream resolution, despite being carried out on a multi-kiloton scale.

Given the industrial relevance of these transformations, the development of sustainable and efficient synthetic pathways for (*R*)-citronellal has emerged as an important challenge in modern chemistry.^10^ Conventional chemical methods often face challenges, such as poor enantioselectivity, harsh reaction conditions, or dependence on hazardous solvents. Enzymatic approaches have therefore emerged as effective, environmentally friendly alternatives that enable stereoselective transformations under mild conditions.^7,10–16^ The challenge so far has been the lack of a biocatalyst that could directly convert citral to enantiopure (*R*)-citronellal, although several engineering attempts have been made, notably with the ene reductases (EREDs) from the flavin mononucleotide (FMN)-dependent Old Yellow Enzyme (OYE) family *Zymomonas mobilis* NCR^17^ and *Saccharomyces cerevisiae* OYE2.^12^ OYEs are known to selectively convert the *E*/*Z* isomers geranial and neral to (*R*)- and (*S*)-citronellal, respectively, depending on their class. Therefore, a notable strategy to obtain (*R*)-citronellal involves the biotransformation of geraniol, a cost-effective and renewable monoterpene, to geranial.

Ribeaucourt *et al.* demonstrated the first one-pot oxidation–reduction system using a copper radical alcohol oxidase (*Cgr*AlcOx) to convert geraniol to geranial, followed by an OYE2-catalyzed asymmetric reduction, achieving (*R*)-citronellal in a high yield and 95.9% *ee* (Scheme 1B).^18^ Although OYEs are now known to be scalable up to 150 g/L,^19^ this study did not have a scalable perspective due to the potential inhibition of *Cgr*AlcOx by the (*R*)-citronellal product. Efforts to immobilize *Cgr*AlcOx to prevent inhibition with the use of organic solvents were promising yet remained at low substrate concentrations.^20^ Following these advances, Zhang *et al.* established a whole-cell biocatalyst for the preparative synthesis of (*R*)-citronellal, reporting both catalytic efficiency and (*R*) stereoselectivity in the reduction of (*E*/*Z*)-citral with an engineered OYE2p from *S. cerevisiae* YJM1341.^12^ Targeted mutagenesis identified Y84 as a key position, with the variant OYE2p_Y84V showing improved activity and increased (*R*) stereoselectivity (Scheme 1B). The substitution of this tyrosine positioned both *E*- and *Z*-citral isomers, geranial and neral, closer to the key hydrogen donor (Y198) and promoted a more favorable (*R*)-selective binding mode.^12^ However, even with the use of whole cells, this system still required external addition of NADH and afforded 95.4% *ee* (*R*)-citronellal (Scheme 1B). In another study, a two-step cascade starting from 18 mg of geraniol, combining a short-chain dehydrogenase (*Aa*SDR1) with the ene reductase variant OYE2_Y83V, enabled 78% conversion and 99.4% *ee* through a biphasic system with *n*-heptane that mitigated general isomerization.^21^ Together, these studies show the versatility of enzymatic oxidation–reduction cascades while also highlighting several bottlenecks, including enzyme inhibition, scalability challenges, and undesired overreduction of the target product. In comparison, this study combines a one-pot cascade at 100 mM geraniol, exceeding substrate loading previously reported while also achieving a higher ee value.

One-pot enzymatic cascades have the advantage of starting from inexpensive available substrates and avoiding isolation and purification of intermediates.^22,23^ In this study, an aryl-alcohol oxidase (AAO) was considered for the oxidation step from geraniol to geranial.^24,25^ Because of their wide range of substrates, these flavin adenine dinucleotide (FAD)-dependent enzymes are well-known for being able to yield a wide variety of aldehydes, including bioactive intermediates, polymer precursors, and flavor and fragrance compounds.^26–28^ AAO from *Streptomyces hiroshimensis* (*Sh*AAO) was recently fully characterized, exhibiting remarkable activity toward structurally diverse alcohols with a preference for long-chain allylic substrates.^29^ Additionally, the potential of *Sh*AAO in scalable synthetic chemistry has been demonstrated through preparative-scale biotransformations at high substrate loadings and through its integration with organocatalytic processes to access chiral synthons.^30^ The capacity of *Sh*AAO to generate geranial from geraniol with a turnover number (TON) of >35 600 for 40 mM geraniol^30^ offered a robust starting point for subsequent stereoselective reduction by the variant OYE2_Y83V, thereby enabling a scalable, fully enzymatic pathway to enantiopure (*R*)-citronellal. Building on this concept, we developed and implemented this AAO–OYE cascade under high substrate loading (Scheme 1B).

We first established the individual steps of the enzymatic cascade and produced and purified FAD-dependent *Sh*AAO as previously described^30^ to catalyze the oxidation reaction of geraniol to geranial. This enzyme uses molecular oxygen as an electron acceptor, which is subsequently reduced to hydrogen peroxide; thus, we supplemented the reaction with catalase for dismutation to water and oxygen (Figure 1). Reactions were carried out to explore intensification with a higher geraniol concentration from 20 to 100 mM (Figure 1A). At 20 mM, we obtained 92% conversion of geraniol, interestingly with different ratios of geranial/neral, 93:7 and 96:4, when using 2.2 and 4.4 μM *Sh*AAO, respectively. A similar trend was observed with higher geraniol concentrations. This effect may be related to differences in the oxidative environment resulting from varying enzyme concentrations and reaction rates or isomerization of geranial to neral.^31^ The underlying cause remains unclear and will require further investigation. At 100 mM geraniol, only 20% was converted, with no increase in conversion when doubling the *Sh*AAO concentration, indicating that the reaction was limited by insufficient oxygen availability in the 2 mL safe-lock Eppendorf tubes. To assess the effect of oxygen, larger reaction vessels were subsequently evaluated.

**Figure 1:**
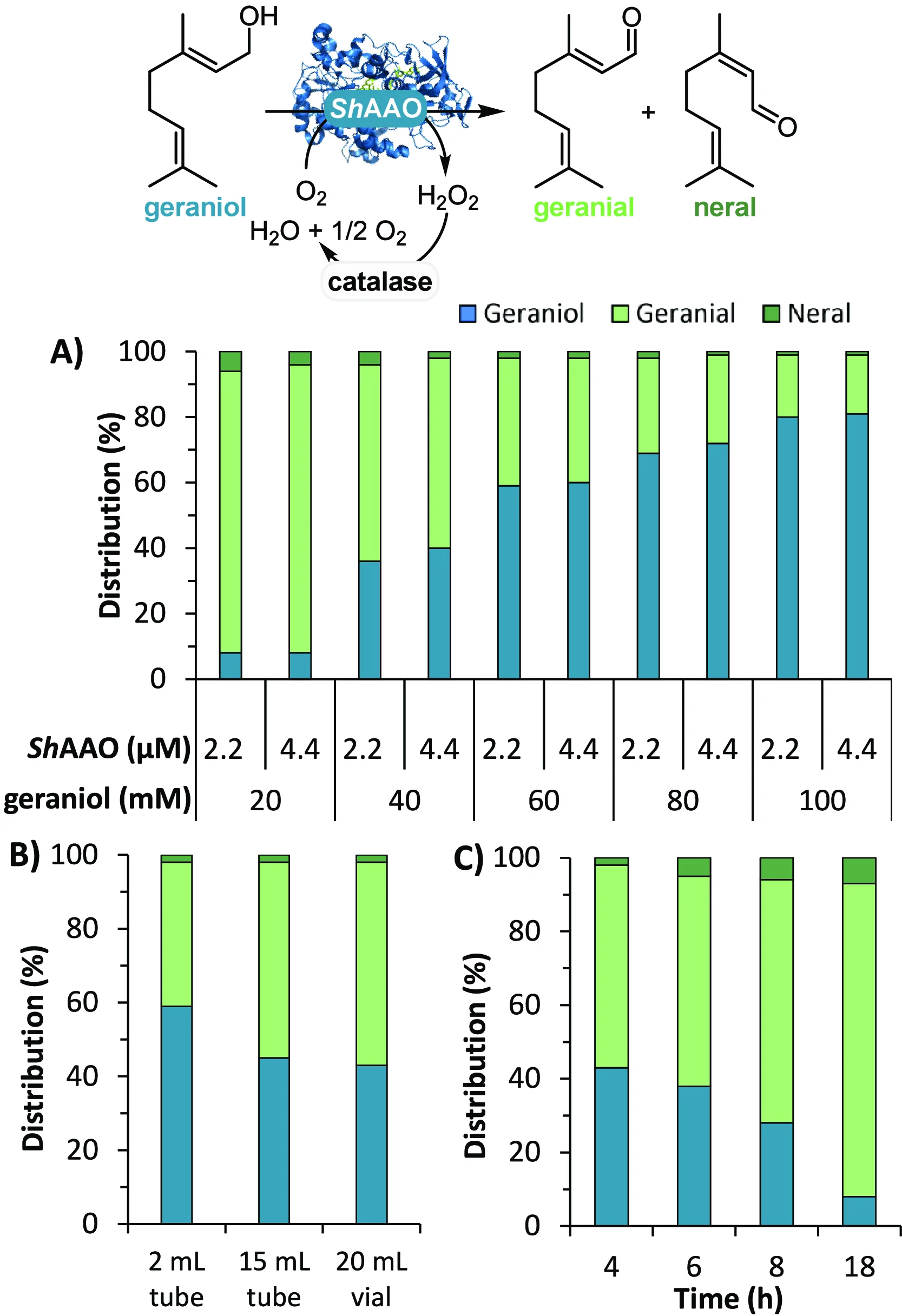
*Sh*AAO-catalyzed geraniol oxidation to geranial. (A) Varying geraniol and *Sh*AAO concentrations; conditions: in a 2 mL safe-lock Eppendorf tube with a final volume of 1 mL containing 50 mM KP*i* buffer at pH 8.0, 20–100 mM geraniol [from a 1 M stock in DMSO, final 2–10% (v/v) DMSO], 1000–2500 U/mL catalase, 2.2 or 4.4 μM *Sh*AAO, 30 °C, 900 rpm, 4 h. (B) 4 h reactions in a 2 mL plastic tube, 15 mL Greiner tube, or 20 mL glass vial; conditions: in 1 mL, 50 mM KP*i* at pH 8.0, 60 mM geraniol, 6% (v/v) DMSO, 1000–2500 U/mL catalase, 2.2 μM *Sh*AAO, 30 °C, 900 rpm. (C) 4–18 h in a 20 mL glass vial.

Reactions with 60 mM geraniol and 2.2 μM *Sh*AAO were conducted in larger reaction vessels from 2 mL (50% headspace) to 15 mL (93% headspace) and 20 mL (95% headspace). Conversions of geraniol increased from 41 to 57% within 4 h (Figure 1B). This enhancement was attributed to the increased headspace and surface area, which facilitated aeration and thus maintained a sufficient oxygen supply for sustained catalysis. Based on these observations, all subsequent reactions were performed in 20 mL glass vials. Under these conditions, a time course experiment using 60 mM geraniol for 18 h and 2.2 μM *Sh*AAO resulted in 92% conversion of geraniol, affording 85% geranial, corresponding to a TON of 25 091 and a turnover frequency (TOF) of 1394 h^–1^ (Figure 1C), which is 1.4-fold higher than previously reported copper-containing alcohol oxidase *Cgr*AlcOx.^18^

For the second step of the cascade, we first explored asymmetric reduction of (*E*/*Z*)-citral using the engineered OYE2_Y83V variant previously reported with an enhanced specific activity of 1.38 U/mg.^21,32^ This variant exclusively converts geranial to (*R*)-citronellal and neral to preferentially (*R*)-citronellal and to a lesser extent (*S*)-citronellal.^12,21,32^ A time-course experiment was performed at 30 °C (Figure 2). After 1 h, 95.8% *ee* (*R*)-citronellal was achieved, with geranial mostly converted and with 28% neral remaining. After 4 h, 99% conversion was achieved, affording 91% (*R*)-citronellal with 91.5% *ee*, demonstrating the efficiency and stereoselectivity of OYE2_Y83V in the biocatalytic reduction of citral, yet the conversion of neral still lowered the overall *ee* value.

**Figure 2:**
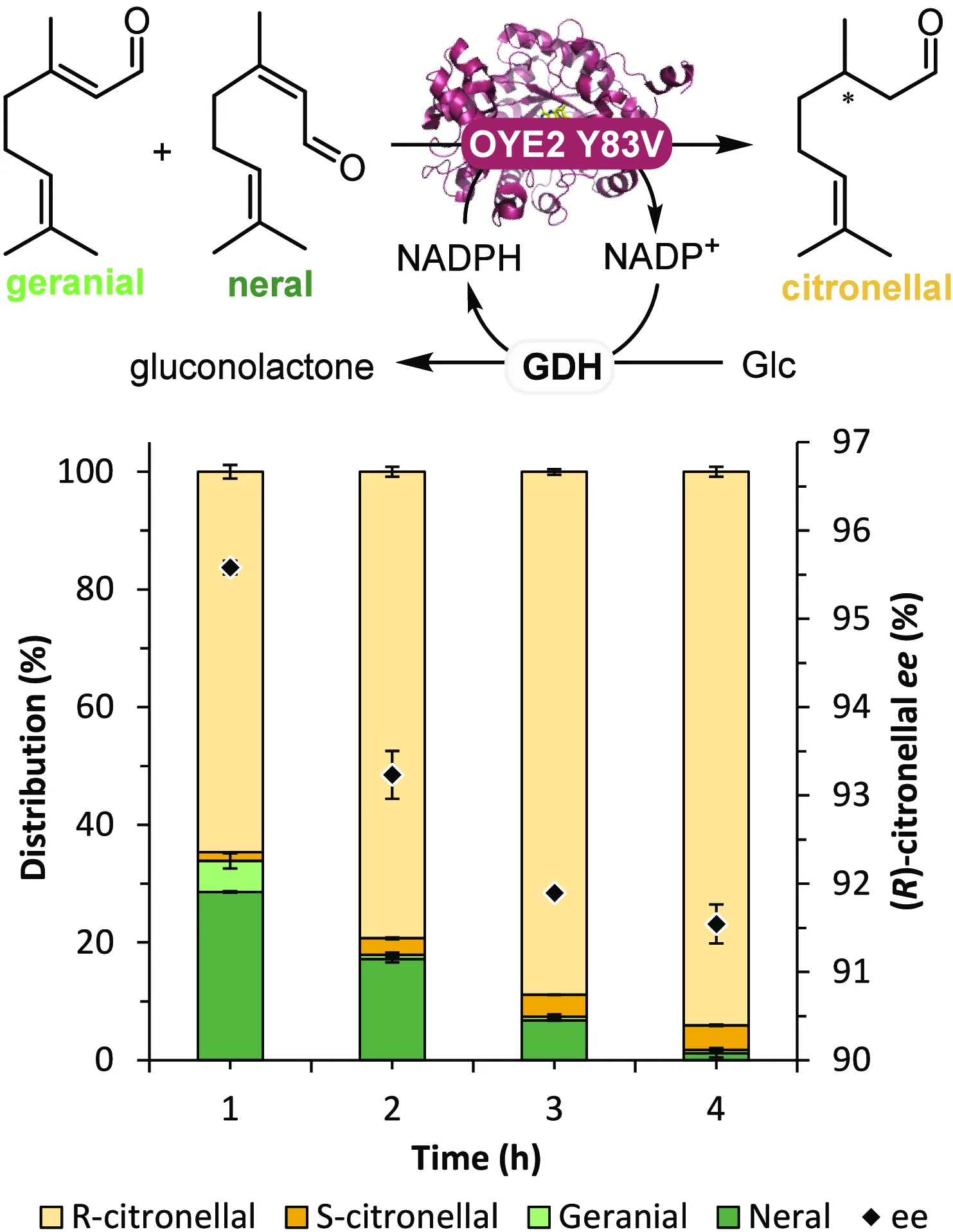
OYE2-catalyzed citral asymmetric reduction to (*R*)-citronellal. Conditions: in 1 mL, 50 mM KP*i* at pH 8.0, 10 mM citral, 1% (v/v) DMSO, 1 mM NADP^+^, 10 U/mL *Bs*GDH, 50 mM glucose (Glc), 5.2 μM OYE2_Y83V, 1–4 h, 30 °C, and 900 rpm in a 2 mL safe-lock Eppendorf tube.

The biocatalytic steps were combined to assess the efficiency of the one-pot cascade. Selective oxidation of geraniol to geranial by *Sh*AAO enabled OYE2_Y83V to reduce geranial to (*R*)-citronellal, with only trace amounts of neral that led to <0.5% (*S*)-citronellal, enabling >99% ee (*R*)-citronellal (Figure 3). The cascade was tested with increasing geraniol concentrations ranging from 10 to 100 mM, varying the cofactor NAD­(P)^+^ (0.2–1 mM) and GDH (1–10 U/mL) concentrations, and conducted for 4 h with 10 mM or 24 h with 100 mM substrate. Reactions with NADPH regeneration consistently gave higher conversion and ee values of (*R*)-citronellal, particularly at 100 mM substrate, whereas NADH recycling minimized the overreduction to (*R*)-citronellol. The higher conversions could be ascribed to the preference of OYE2 for NADPH over NADH, leading to higher yields in 4 h, whereas the conversion to citronellol can be explained by GDH promiscuous activity, with a cofactor preference for NADPH.^20,33^ Variation in GDH loading had little impact on the overall reaction (Figure 3). At 100 mM geraniol, 91% (*R*)-citronellal was obtained with 99.2% *ee* with NADPH recycling.

**Figure 3:**
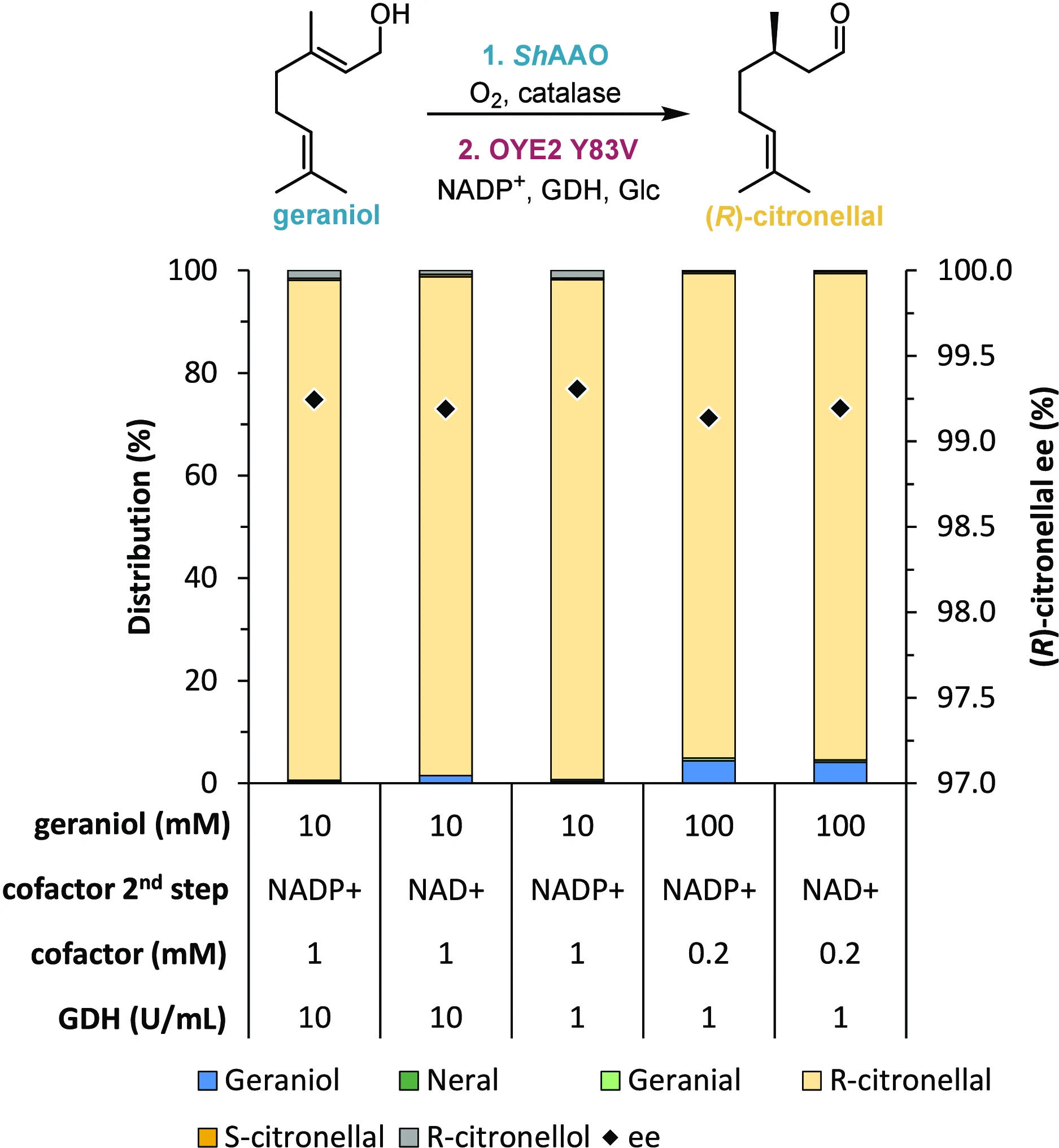
Biocatalytic (*R*)-citronellal production from geraniol. Conditions: in 1 mL, 50–100 mM KP*i* at pH 8.0, 10–100 mM geraniol, 1% (v/v) DMSO (10 mM reactions), 1000–2500 U/mL catalase, 2.2 μM *Sh*AAO (100 mM reactions) or 5.2 μM *Sh*AAO (10 mM reactions), 5.2 μM OYE2_Y83V, 0.2–1 mM NAD­(P)^+^, 1–10 U/mL *Bs*GDH, 50–200 mM Glc in a 20 mL glass vial at 30 °C, 900 rpm, 4 h. Scale up (100 mM geraniol): 24 h.

To further evaluate the scalability of this cascade, the one-pot reaction was carried out on a 1 mmol scale (100 mM geraniol, 10 mL volume, 15.4 g/L), achieving 90% conversion to (*R*)-citronellal and 99.8% *ee*. After column chromatography purification (95:5 *n*-pentane/EtOAc) to remove unreacted geraniol, 95.0 mg of (*R*)-citronellal was isolated (61% yield and 98% purity; Figures S22 and S26) with an *ee* of >99.8%, representing the highest enantiopurity reported to date. The difference between the 90% conversion and the isolated yield is likely attributed to product loss during purification and solvent removal under reduced pressure given the high volatility of (*R*)-citronellal.

To avoid using the GDH cofactor recycling system, we envisioned coupling an electron mediator that would be reduced by flavin adenine dinucleotide (FAD) of AAO and accepted by the OYE to reduce its FMN prosthetic cofactor (Figure 4). First, *Sh*AAO was screened with a wide range of electron mediators under anaerobic conditions (Figure 4 and Figure S6), obtaining 99% conversion of 10 mM geraniol with methylene blue.

**Figure 4:**
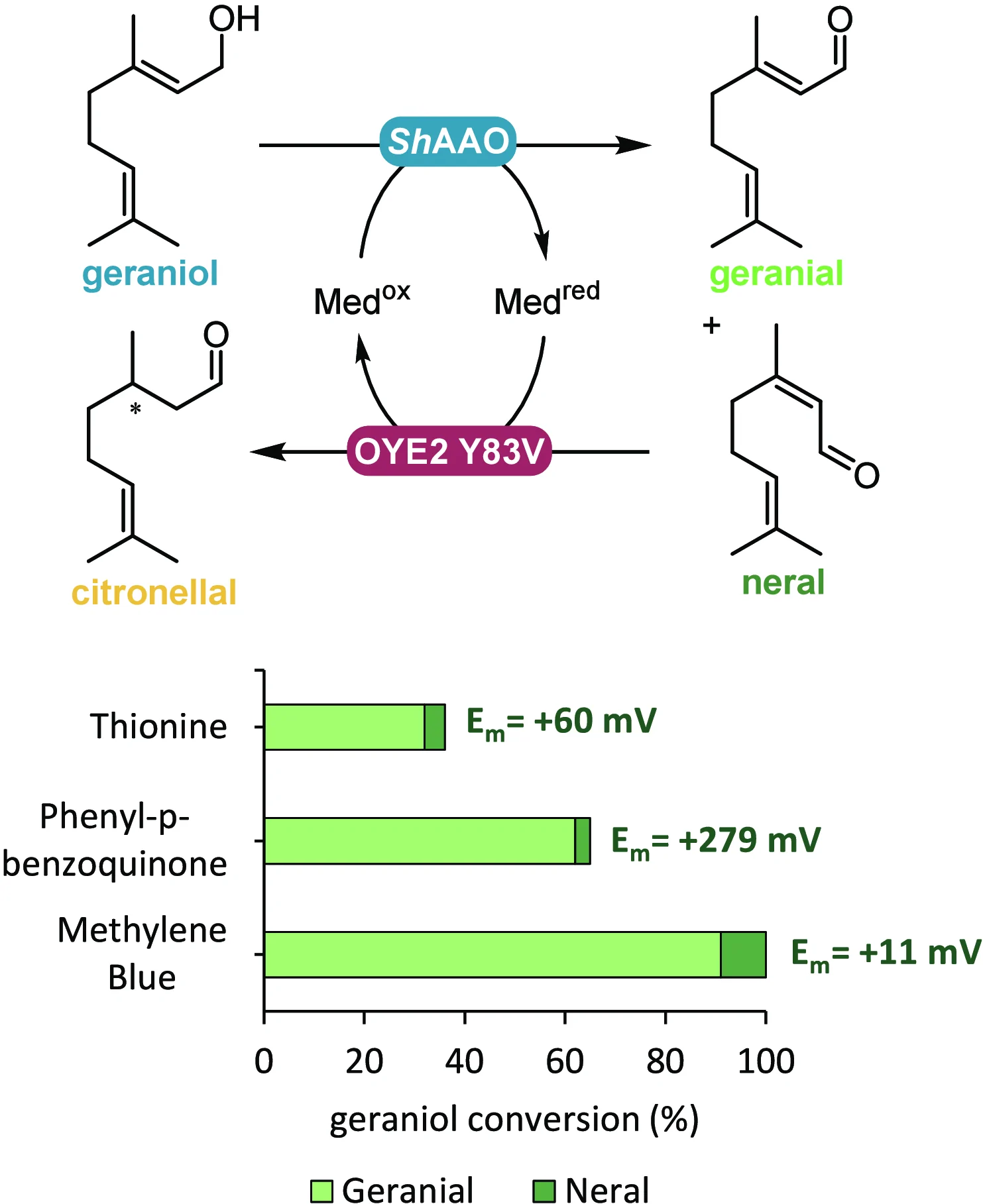
Coupled enzymatic cascade from geraniol to citronellal with an electron mediator (Med) bypassing NAD­(P)H cofactor recycling. Conditions: in 1 mL, 50 mM KP*i* at pH 8.0, 50 mM electron mediator, 2.2 μM *Sh*AAO, 5 μM OYE2_Y83V, 10 mM geraniol, at 30 °C and 900 rpm for 4 h under an anaerobic atmosphere in a Coy chamber.

Then, OYE2_Y83V was coupled with *Sh*AAO, and the cascade was tested with each of the best three mediators (Figure S7). Unfortunately, only geraniol oxidation to geranial and neral was observed, and no reduction to citronellal was obtained. This suggests that mediators accepted by one enzyme were not accepted by the other, potentially due to either substrate acceptance or redox potential differences. Further studies of this cascade with other enzymes and electron mediators are ongoing.

In conclusion, we established a one-pot biocatalytic cascade by combining two compatible enzymes, *Sh*AAO and OYE2_Y83V, enabling the efficient and selective transformation of the inexpensive substrate geraniol into enantiopure (*R*)-citronellal. Additionally, this system tolerated high substrate loadings at 100 mM, achieving 95 mg of isolated product and 99.8% *ee*, representing the highest enantiomeric excess reported for (*R*)-citronellal to date. These results highlight both the efficiency of the cascade and potential for scale-up application. Future perspective of this cascade could be to bypass the cofactor recycling by using an electron mediator. Enantiopure (*R*)-citronellal can be further cyclized by terpene cyclases to valuable terpenes, such as (−)-menthol^34^ or the insect repellent *p*-menthane-3,8-diol (PMD),^32^ to implement a sustainable synthetic process.

## Supplementary Material



## Data Availability

The data underlying this study are available in the published article and its Supporting Information.

## Notes

All experimental details, including enzyme production and purification, reaction protocols, GC analyses, and NMR spectrum (PDF)
