# Supplementary material for: Biocatalytic Cascade from Geraniol to Enantiopure (R)‑Citronellal Using an Aryl-Alcohol Oxidase and Ene Reductase
Source: Org Lett. 2026 Sep 9;28(37):11469–74. doi: 10.1021/acs.orglett.6c02985 (PMC13595905; doi:10.1021/acs.orglett.6c02985)
Supplement: Supplementary file 1 [file ol6c02985_si_001.pdf]

Supporting information

Biocatalytic cascade from geraniol to enantiopure (*R*)-citronellal using an aryl-alcohol oxidase and ene reductase

Claudia Ferrer-Carbonell,[ǂ,a] Christian Ascaso-Alegre,[ǂ,a,b] Paula Cinca-Fernando,[b,c] Patricia Ferreira,[b,c] Juan Mangas-Sánchez,[d,*] Caroline E. Paul[a,*]
ǂ equal first authors

[a] Biocatalysis section, Department of Biotechnology, Delft University of Technology, Van der Maasweg 9, 2629 HZ Delft, The Netherlands; c.e.paul@tudelft.nl
[b] Departamento de Bioquímica y Biología Molecular y Celular, Facultad de Ciencias, Universidad de Zaragoza, Zaragoza, Spain
[c] Instituto de Biocomputación y Física de Sistemas Complejos, BIFI (GBsC-CSIC Joint Unit), Universidad de Zaragoza, Zaragoza, Spain
[d] Department of Organic and Inorganic Chemistry, IUQEM, University of Oviedo, Julián Clavería 8, Oviedo 33006, Spain; mangasjuan@uniovi.es

Contents

General information 2
Enzyme production, purification and characterisation 2
  Aryl-alcohol oxidase *Sh*AAO 2
Biocatalytic reactions 3
  AAO-catalysed oxidation of geraniol 3
  OYE-catalysed reduction of geranial 4
  AAO-OYE cascade from geraniol to (*R*)-citronellal 4
  Data tables 8
Electron mediators for neutral redox system 9
GC analyses 11
GC chromatograms 12
  GC Method 1 12
  GC Method 2 13
  GC Method 3 16
Calibration curves 17
NMR spectra 18
References 19

General information

Chemicals were obtained from Merck Sigma-Aldrich, TCI Chemicals, abcr GmbH in the highest purity available and used as received. In particular, chemicals and enzymes obtained from Merk Sigma-Aldrich: geraniol (>98%, CAS 106-24-1), citral (>95%, CAS 5392-40-5), (*R*)-(+)-citronellal (CAS 2385-77-5), (*S*)-(–)-citronellal (CAS 5949-05-3), (*R*)-(+)-citronellol (CAS 1117-61-9), (*S*)-(–)-citronellol (CAS 7540-51-4), cOmplete™ mini EDTA-free protease inhibitor pills, catalase from bovine liver (C9322, 2000-5000 U/mg), flavin mononucleotide sodium salt (FMN, CAS 6184-17-4) and deoxyribonuclease I crude lyophilized (DNase I, CAS 9003-98-9). Isopropyl-β-D-thiogalactopyranoside (IPTG, ≥99%, CAS 367-93-1) was obtained from CliniSciences. The reduced form of β-nicotinamide adenine dinucleotide phosphate (NADPH, CAS 2646-71-1) was purchased from Oriental Yeast Co (OYC). The oxidized form $NADP^+$ (97.6%, CAS 698999-85-8) and commercial NAD(P)H oxidase (NOx, CAS 77106-92-4) were sourced from Prozomix Ltd (UK).

Cells were lysed through a Multi Cycle Cell Disruptor from Constant Systems Ltd at 4 °C. Immobilised metal (nickel)-affinity chromatography (IMAC) purification was performed on a Next Generation Chromatography (NGC) system from Bio-Rad using 5 mL GE HealthCare HisTrap FF Crude columns unless otherwise specified.

Biocatalytic reactions were incubated in an Eppendorf ThermoMixer C with a top lid at 30 °C and 900 rpm unless otherwise stated.

Gas chromatography (GC) was performed on Shimadzu GC-2010 gas chromatographs (Shimadzu Corporation, Kyoto, Japan) equipped with a flame ionisation detector (FID), and achiral and chiral columns. Products were confirmed with reference standards. Product concentrations were obtained with calibration curve equations using 5 mM dodecane as an internal standard in ethyl acetate (EtOAc) used to extract all compounds.

Nuclear magnetic (NMR) spectroscopy was carried out on an Agilent 400 MHz (9.4 Tesla) spectrometer operating at 399.67 MHz for ^1H at 298 K. Spectra were interpreted using the software MestReNova (version 12.0.1 by Mestrelab Research S.L.).

Enzyme production, purification and characterisation

Old Yellow Enzyme 2 from *Saccharomyces cerevisiae* (*Sc*OYE2, accession number Q03558) single mutant Y83V (OYE2 Y83V) was recombinantly produced in *Escherichia coli* BL21 Gold(DE3) cells using the plasmid pET-28b(+)-*oye2_Y83V* and purified as previously described.[1] Glucose dehydrogenase from *Bacillus subtilis* double mutant *Bs*GDH E170K_Q252L was recombinantly produced in *E. coli* BL21 Gold(DE3) cells with the plasmid pET-28a(+)-*bsgdh_E170K_Q252L* and purified as previously described.[2]

Aryl-alcohol oxidase *Sh*AAO

Aryl-alcohol oxidase from *Streptomyces hiroshimensis* (*Sh*AAO, accession number WP_190019735.1) was recombinantly produced in *E. coli* BL21 C41(DE3) cells with the plasmid pET-28a-*Sh*AAO and purified as previously described, affording a functional active enzyme.[3]

The storage stability was evaluated by performing thawing and refreezing cycles on the enzyme. Then, its conversion and geranial ratio were assessed in the geraniol oxidation reaction. The experimental

conditions for this assay were as follows: a final volume of 1.0 mL, comprising 10 mM geraniol (from a 1 M DMSO stock, 1 % v/v), 1000-2500 U/mL catalase and 1.1 µM *Sh*AAO.

Following the initial reaction, the enzyme solution underwent flash-freezing with liquid nitrogen, followed by a 15 min storage period at -20 °C before thawing at room temperature (20 °C). This cycle was repeated three times, as outlined in Figure S1. After each cycle, no discernible difference was observed in either conversion or geranial ratio. This suggests that the AAO exhibits stability even after being subjected to freezing and thawing for at least three cycles. However, when kept in the fridge (4 °C) for longer periods of time (3 days), precipitation of the enzyme was observed, leading to a slight loss of activity.

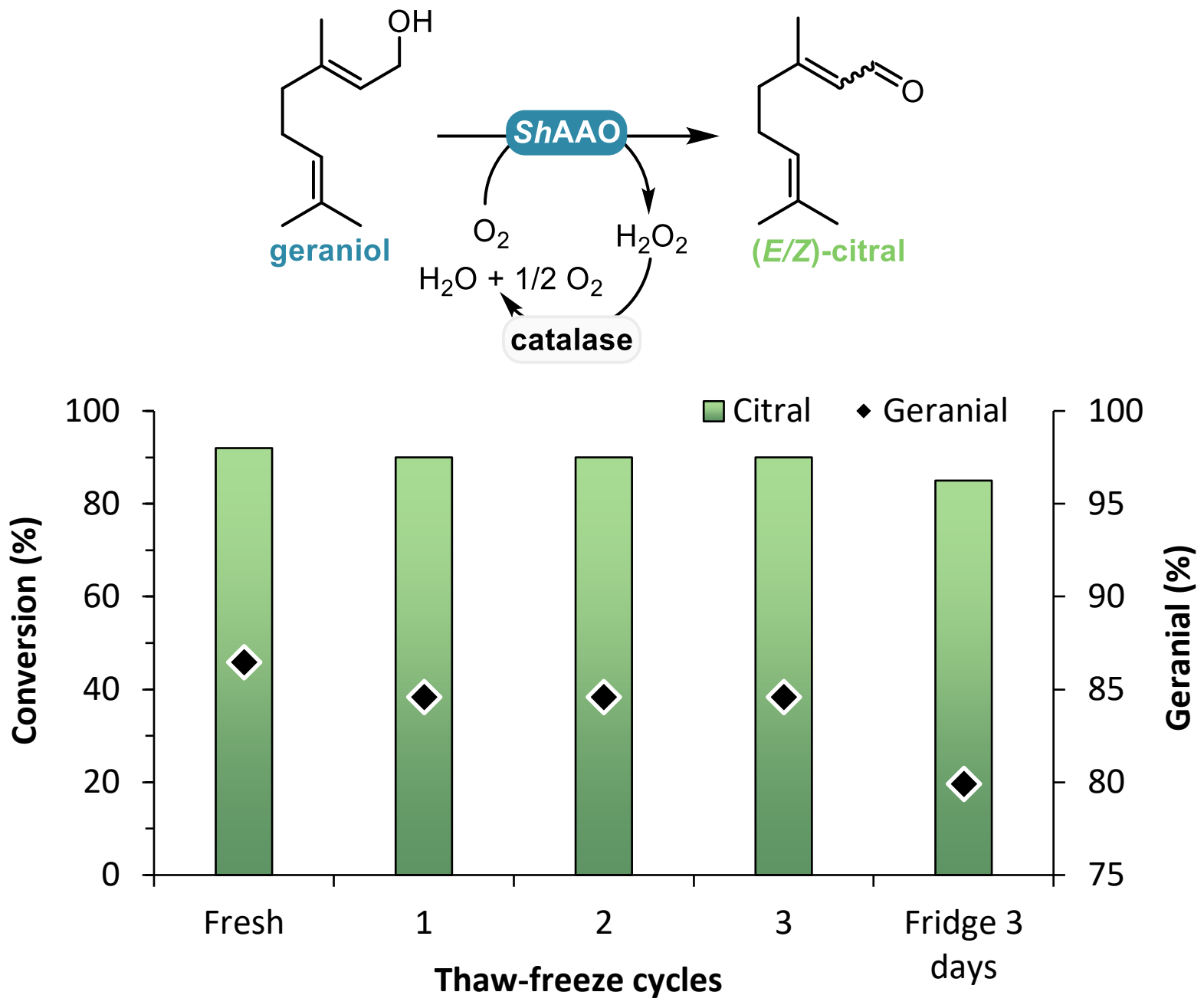

Figure S1. Stability assay. Geraniol to geranial conversion and geranial:neral ratio after 0-3 thaw-freeze cycles of *Sh*AAO and after storage at 4 °C for 3 days.
Conditions: in 1 mL, 50 mM KP*i* pH 6.0, 1000-2500 U/mL catalase, 1.1 µM *Sh*AAO, 10 mM geraniol, 1% v/v DMSO, 30 °C, 900 rpm, 4 h.

Biocatalytic reactions

Biocatalytic reactions were incubated in an Eppendorf ThermoMixer C with a top lid at 30 °C and 900 rpm unless otherwise stated. The shaking speed was taken as 900 rpm based on previous experiments with oxygen-dependent styrene monooxygenase and not optimized.[4] The reaction temperature was set at 30 °C based on the thermal stability of *Sh*AAO (Tm = 48.6 °C), as its activity begins to slightly decrease at 40 °C and steeply drops between 45-50 °C.[5] OYE2 Y83V remains stable at 30 and 40 °C.[1] Therefore 30 °C was selected without further optimization.
After incubation, all the reactions were extracted by adding EtOAc (2 × 0.7 mL) containing 5 mM dodecane as internal standard (IS), vortexing, and centrifuging (14,000 rpm, 3 min, 10 °C). The organic layer was separated, dried with anhydrous $MgSO_4$, and transferred to a GC vial.
Conversion values were calculated by dividing the area of products formed by the total area of all peaks corresponding to the biocatalytic reaction, multiplied by 100.

AAO-catalysed oxidation of geraniol

The oxidation reactions of geraniol to geranial were performed with purified *Sh*AAO. To optimize the reaction conditions, parameters such as varying enzyme concentration and pH were studied (**Figure**

S2). Reactions were carried out in a 1 mL safe-lock Eppendorf containing 50 mM KP*i* at pHs between 6.0 and 8.0, 10 mM geraniol, 1% v/v DMSO, 1000-2500 U/mL catalase, and enzyme concentrations ranging from 0.88 to 2.2 µM. These reactions were carried out for 4 h, at 900 rpm and 30 °C.

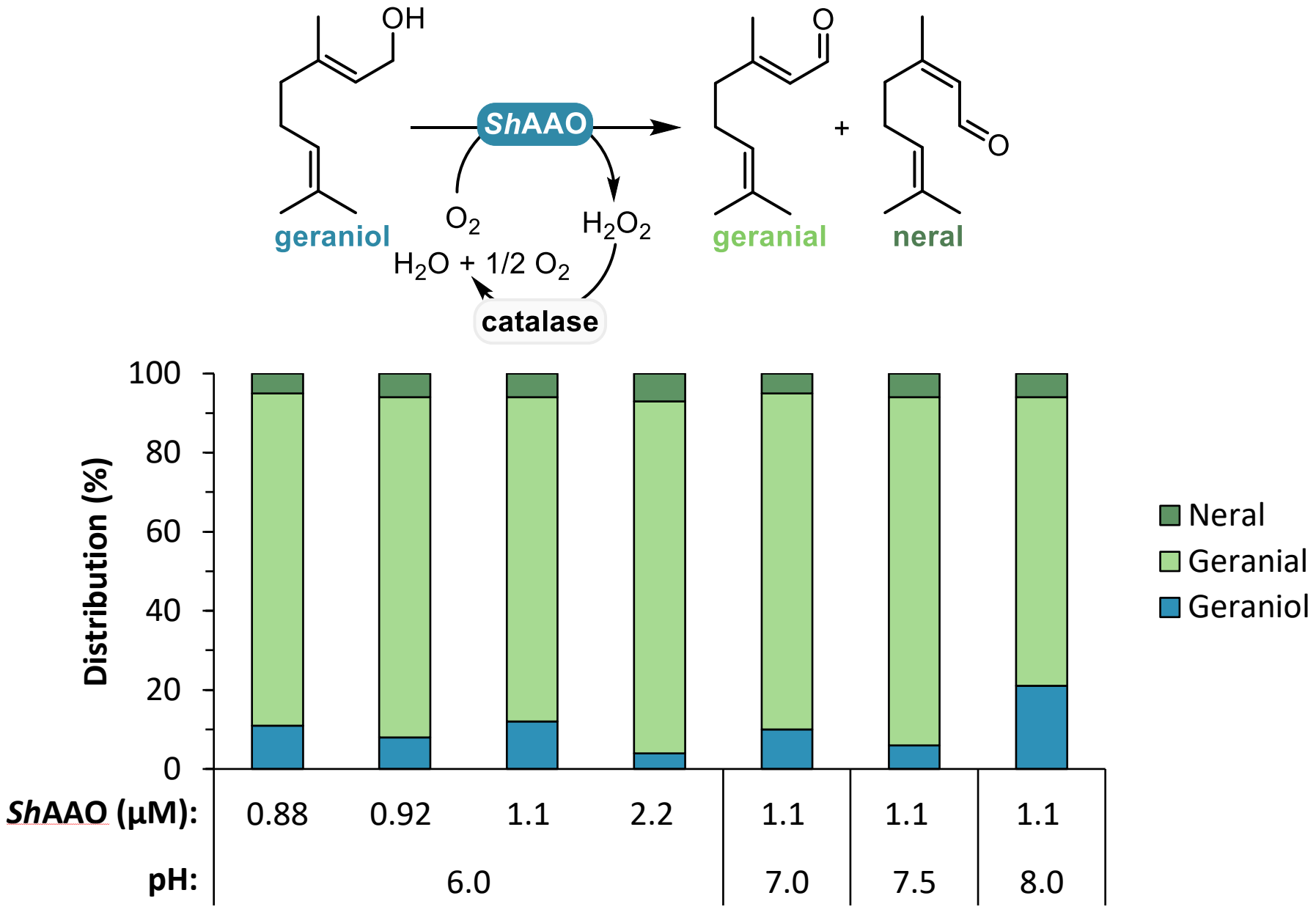

Figure S2. Geraniol oxidation to geranial screening different pHs and *Sh*AAO concentrations. Conditions: in 1 mL, 50 mM KP*i* pH 6.0-8.0, 10 mM geraniol, 1% v/v DMSO, 900 U/mL catalase and 0.88-2.2 µM *Sh*AAO. The reaction mixtures were incubated at 30 °C, 900 rpm, for 4 h.

OYE-catalysed reduction of geranial

The citral reduction reactions took place in 50 mM KP*i* pH 8.0, 10 mM citral, 1% v/v DMSO, 20 mM glucose (Glc), 1 mM $NADP^+$, 10 U/mL *Bs*GDH, and 5 µM OYE2 Y83V, 1 mL volume, 30 °C, 900 rpm, 1 h. Reactions were quenched by cooling on ice, extracted with EtOAc containing 5 mM dodecane as IS, vortexing, and centrifuging (14,000 rpm, 3 min, 10 °C). Finally, the organic layer was separated, dried with anhydrous $MgSO_4$, and transferred to a GC vial.

AAO-OYE cascade from geraniol to (*R*)-citronellal

After optimising both enzymatic steps, they were combined into a one-pot cascade. Reactions were performed in a final volume of 1 mL in a 20 mL reaction vial containing 50 mM KP*i* at different pH values (6.0-8.0), 10 mM geraniol, 1 % v/v DMSO, 1000-2500 U/mL catalase, 2.2 µM *Sh*AAO, 5 µM OYE2 Y83V, 50 mM Glc, 10 U/mL *Bs*GDH and 1 mM $NADP^+$. These reactions were incubated for 4 h at 30 °C and 900 rpm (**Figure S3**). Reactions were quenched by cooling on ice, extracted with EtOAc (2 × 0.7 mL), dried over anhydrous $MgSO_4$ and transferred to GC vials for analysis.

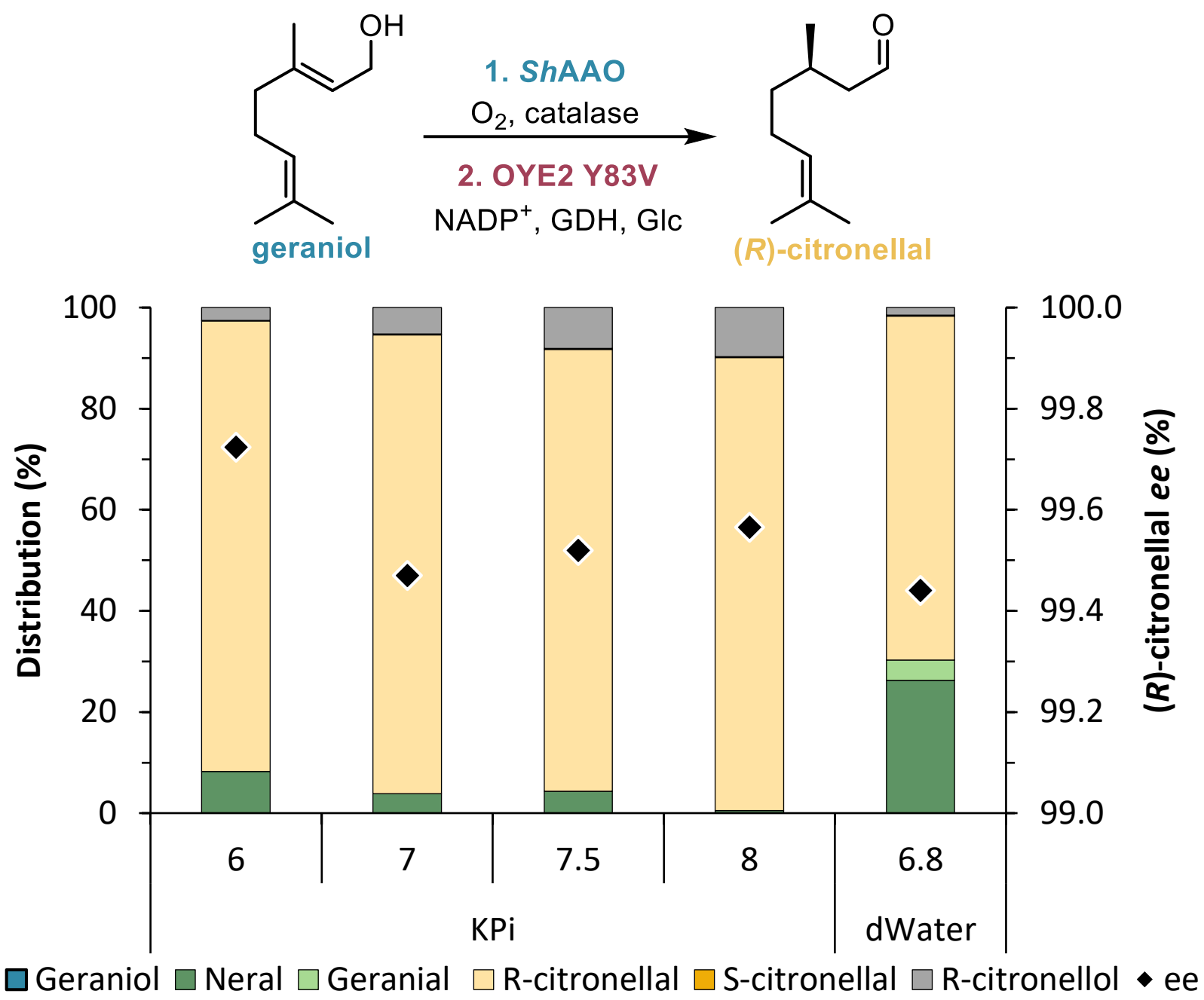

Figure S3. One-pot cascade starting from geraniol at different pH values.
Conditions: in 1 mL, 50 mM KP*i* at pH values ranging 6.0-8.0, 50 mM Glc, 10 U/mL *Bs*GDH, 1 mM NADP+, 1000-2500 U/mL catalase, 2.2 µM *Sh*AAO, 5 µM OYE2 Y83V, 10 mM geraniol, 1 % v/v DMSO, 30 °C, 900 rpm, 4 h.

We observed that reaction conversions remained unaffected by pH between 6.0 and 8.0. In contrast, reactions carried out in distilled water showed reduced performance, yielding an (*R*)-citronellal conversion of 68% compared with the 88-91% in KP*i* buffer.
Because OYE2 showed a higher activity at alkaline pH values, particularly at pH 8.0, KP*i* pH 8.0 was selected as the optimal reaction medium for subsequent experiments.

To prove the scalability of the cascade process, higher concentrations of geraniol (40-100 mM) were tested (**Figure S4**). In this case, reaction times were increased to 24 h to reach the highest conversion.

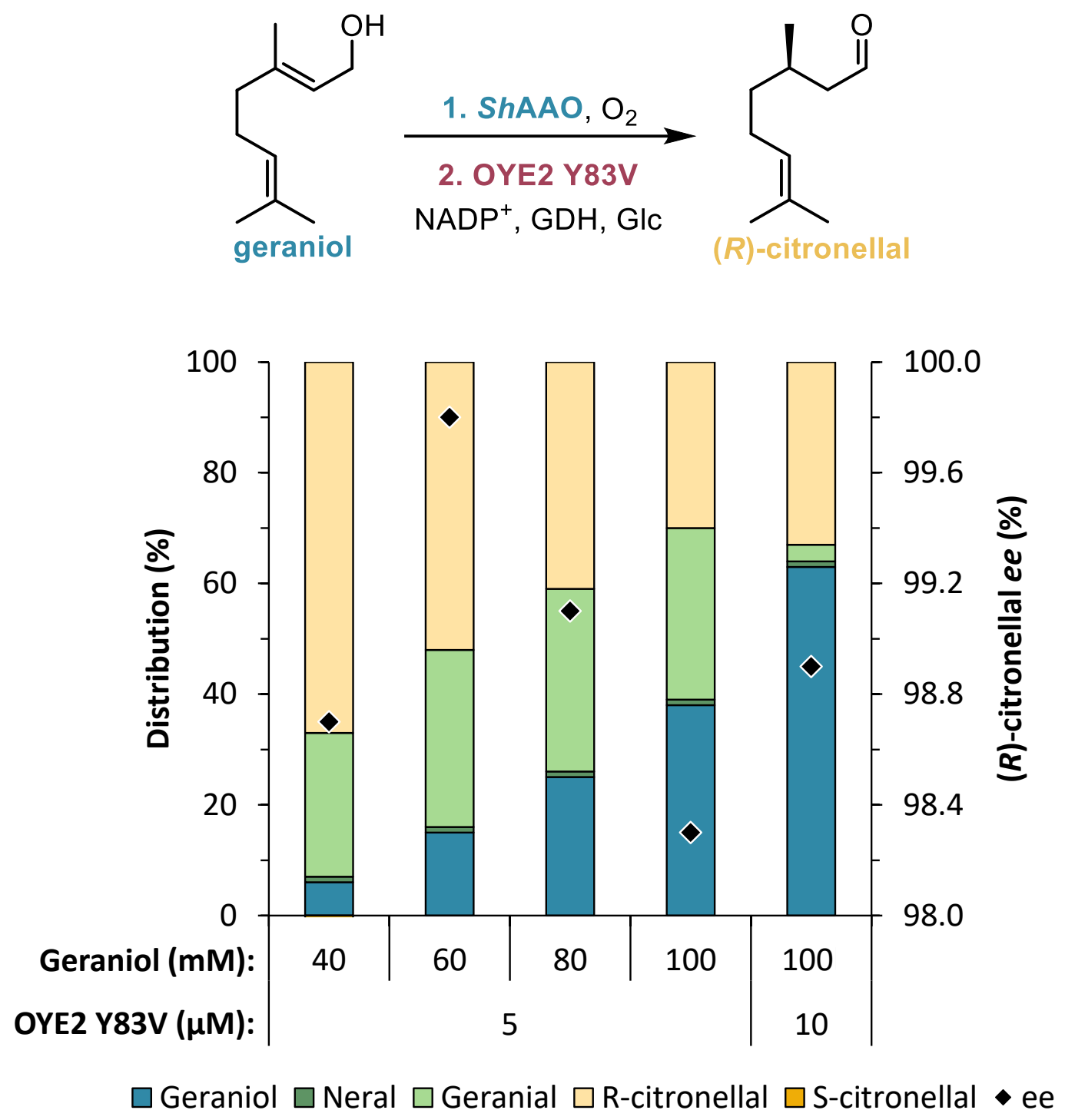

Figure S4. Scale-up of the full cascade starting from geraniol.
Conditions: In 1 mL, 50 mM KP*i* pH 8.0, 200-500 mM Glc, 1 U/mL GDH, 0.2 mM NAD^+, 2.2 µM *Sh*AAO, 5 or 10 µM OYE2 Y83V, 40-100 mM geraniol, 30 °C, 900 rpm, 24 h.

Unreacted geraniol was observed with increased substrate concentrations. This could be due to the *Sh*AAO inhibition by the presence of H_2O_2, as no catalase was added in this set of experiments. Cinca-Fernando and co-workers observed that H_2O_2 concentrations between 30-35 mM inhibited the enzyme after 24 h.[5]

Considering the enzyme inhibition, the presence of catalase was then evaluated at a substrate concentration of 100 mM, which improved the conversion of geraniol oxidation. Nevertheless, complete conversion could not be achieved, which might be attributed to the acidification of the reaction medium due to the formation of a high amount of glucuronolactone. To investigate its potential effect, the ionic strength of the buffer was increased from 50 mM to 100 mM (**Figure S5**).

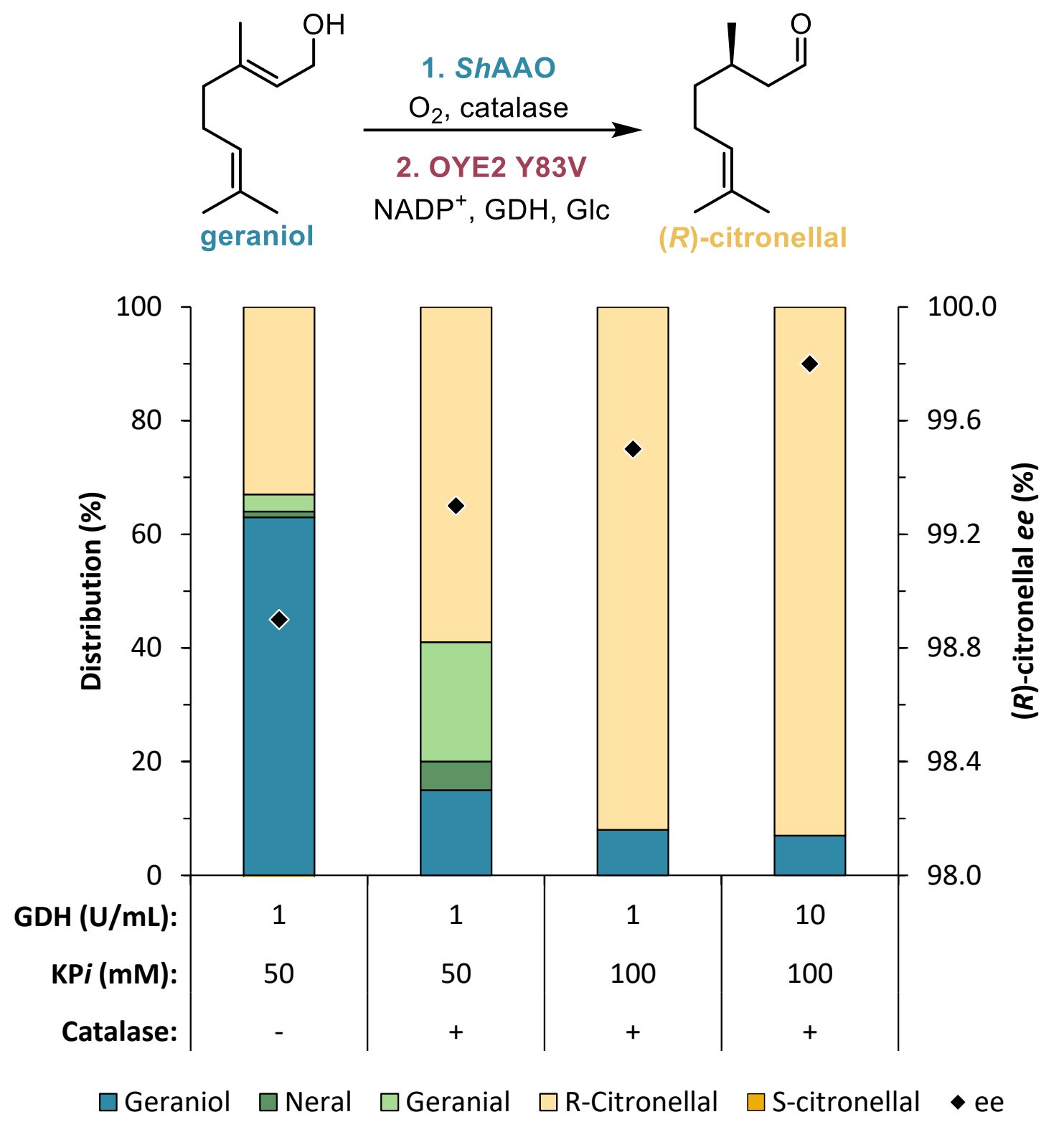

Figure S5. Scale-up cascade reaction optimization.
Conditions: In 1 mL, 50 or 100 mM KP*i* pH 8.0, 100 mM geraniol, 2.2 µM *Sh*AAO, 10 µM OYE2 Y83V, 200 mM Glc, 1 or 10 U/mL GDH, 0.2 mM NAD^+, 0 or 1000-2500 U/mL catalase at 30 °C and 900 rpm for 24 h in a 20 mL glass vial.

This adjustment enabled a final (*R*)-citronellal yield of 92% with 99.5% *ee*, with only 8% geraniol remaining. The GDH concentration was also increased from 1 to 10 U/mL, which resulted in the same (*R*)-citronellal yield but with an improvement in the enantioselectivity, from 99.5 to 99.8% *ee*.

Data tables

Table S1. Reactions for the optimization of the cofactor recycling system with $NADP^+$ or NAD^+.

Entry	[Geraniol] (mM)	DMSO v/v (%)	[*Sh*AAO] (µM)	[Catalase] (U/mL)	[OYE2_Y83V] (µM)	Cofac-tor	[Cofactor] (mM)	[*Bs*GDH] (U/mL)	[Glc]	Buffer	Time (h)	Conv. (%)	(*R*)-citron-ellal (%)	(*S*)-citron-ellal (%)	(*R*)-citronel-lol (%)	*ee* (%)
1	10	1	2.2	1000-2500	5	$NADP^+$	1	10	50	50 mM KP*i* pH 8.0	4	>99	89	0.2	9	99.6
2	10	1	2.2	1000-2500	5	NAD^+	1	10	50	50 mM KP*i* pH 8.0	4	>99	94	0.2	5	99.5
3	10	1	2.2	1000-2500	5	NAD^+	1	1	50	50 mM KP*i* pH 8.0	4	>99	93	0.1	2	99.7
4	10	1	2.2	0	5	NAD^+	1	1	50	50 mM KP*i* pH 8.0	4	>99	91	0.1	0	99.7
5	10	1	2.2	0	5	NAD^+	0.2	1	50	50 mM KP*i* pH 8.0	4	>99	94	0.1	0	99.7

Conditions: buffer KP*i* pH 8.0, 50 mM glucose, 2.2 µM *Sh*AAO, 5 µM OYE2_Y83V, 10 mM geraniol (with 1% v/v DMSO from a stock of 1 M), 1 mL volume, 4 h

Table S2. Reactions for the optimization of the scale up using NAD^+ as cofactor.

Entry	[Geraniol] (mM)	DMSO v/v (%)	[*Sh*AAO] (µM)	[Catalase] (U/mL)	[OYE2_Y83V] (µM)	[Cofactor] (mM)	[*Bs*GDH] (U/mL)	[Glc]	Buffer	Time (h)	Volume (mL)	Conv. (%)	(*R*)-citron-ellal (%)	(*S*)-citron-ellal (%)	*ee* (%)
1	10	1	1.1	0	5	0.5	1	50	50 mM KP*i* pH 8.0	4	1	>99	91	0.1	99.7
2	10	1	1.1	0	5	0.2	1	50	50 mM KP*i* pH 8.0	4	1	>99	94	0.1	99.7
3	40	0	2.2	0	5	0.2	1	200	50 mM KP*i* pH 8.0	24	1	94	67	0.4	98.7
4	60	0	2.2	0	5	0.2	1	300	50 mM KP*i* pH 8.0	24	1	85	52	0.3	99.8
5	80	0	2.2	0	5	0.2	1	400	50 mM KP*i* pH 8.0	24	1	75	41	0.2	99.1
6	100	0	2.2	0	5	0.2	1	500	50 mM KP*i* pH 8.0	24	1	72	30	0.2	98.3
7	100	0	2.2	0	10	0.2	1	500	50 mM KP*i* pH 8.0	24	1	37	33	0.2	98.9
8	100	0	4.4	1000-2500	10	0.2	1	500	50 mM KP*i* pH 8.0	24	1	85	59	0.2	99.3
9	100	0	4.4	1000-2500	10	0.2	10	500	50 mM KP*i* pH 8.0	24	1	74	60	0.2	99.3
10	100	0	4.4	1000-2500	15	0.2	10	500	50 mM KP*i* pH 8.0	24	1	77	70	0.1	99.7
11	100	0	4.4	1000-2500	10	0.2	1	200	100 mM KP*i* pH 8.0	24	1	92	92	0.2	99.5
12	100	0	2.2	1000-2500	10	0.2	10	200	100 mM KP*i* pH 8.0	24	1	93	93	0.1	99.8
13	100	0	2.2	1000-2500	10	0.2	10	200	100 mM KP*i* pH 8.0	24	10	92	90	0	>99.9[a]

[a] isolated purified product, see Figure S22.

Electron mediators for neutral redox system

The development of a neutral redox system would avoid the use of the cofactor recycling system, thereby greatly reducing cost and reaction medium issues such as acidification from the gluconic acid. Previous studies showed that OYE could be reduced by electron mediators such as safranin, phenosafranine, methyl viologen,[6] and reduced FMN.[7, 8]
First, a screening of electron mediators for the *Sh*AAO was performed to establish those accepted by the enzyme, reduced by the $FADH_2$, thus regenerating the FAD without the need for oxygen. The screening was performed under anaerobic conditions using a wide variety of mediators (**Figure S6**).

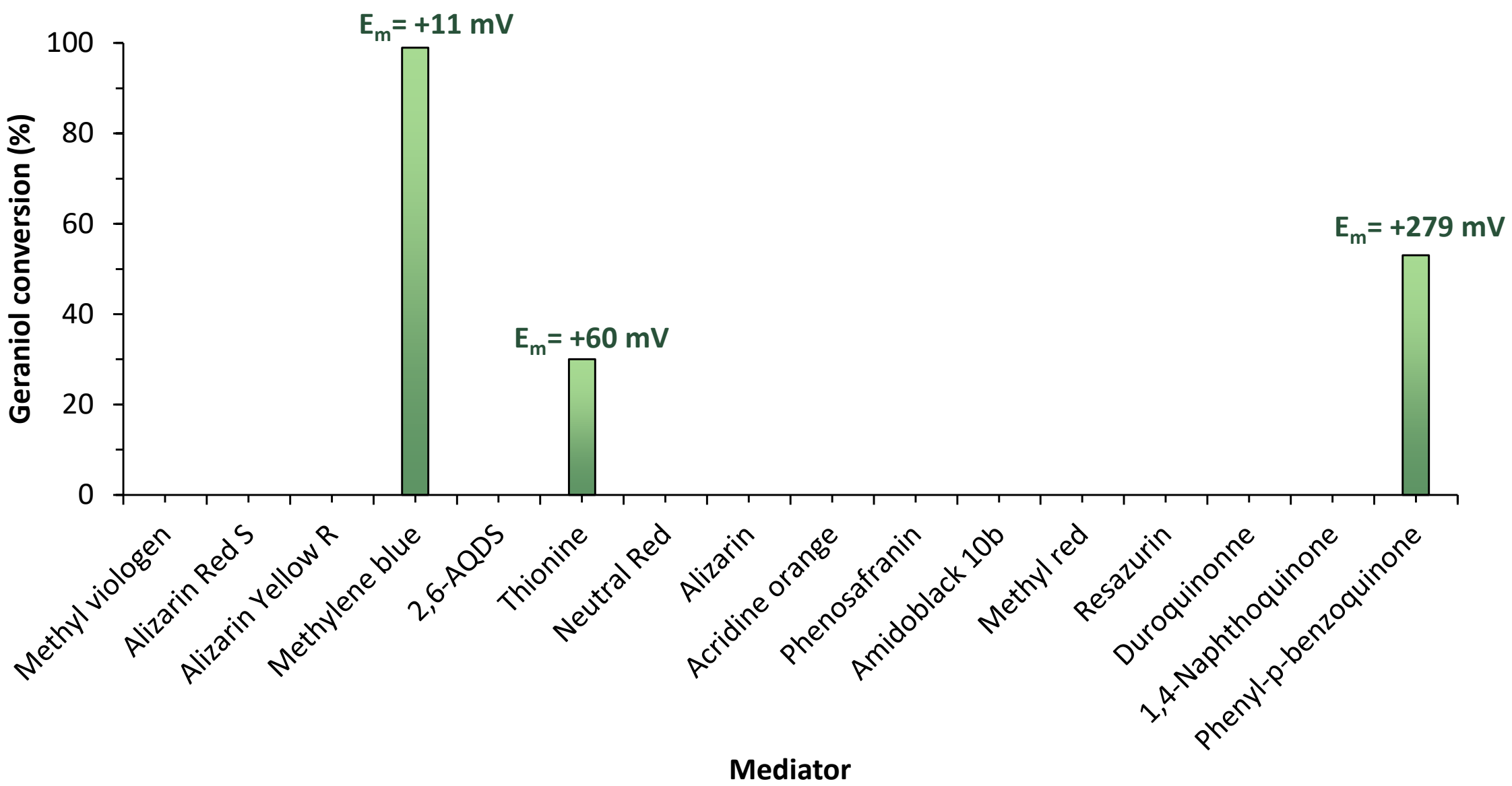

Figure S6. Electron mediator screening with *Sh*AAO.
Conditions: in 1 mL, 50 mM KP*i* pH 8.0, 50 mM electron mediator, 1000-2500 U/mL catalase, 2.2 µM *Sh*AAO and 10 mM geraniol at 30 °C and 900 rpm for 4 h under anaerobic atmosphere in a Coy chamber. Redox potentials for thionine [9] and phenyl-*p*-benzoquinone[10] were previously published.

Three mediators were found to be capable of being used as electron acceptors by *Sh*AAO during geraniol oxidation, with methylene blue providing the best results, achieving 99% conversion of geraniol, followed by phenyl-*p*-benzoquinone (53%) and thionine (30%). These mediators were subsequently tested, both in catalytic amounts and in excess, in an attempt to combine the *Sh*AAO with OYE2_Y83V. However, no traces of (*R*)-citronellal were observed under any condition; only neral and geranial were observed (Figure S7). This lack of coupling is due to a mismatch in mediator acceptance between the two enzymes: known electron acceptors by OYEs, such as phenosafranin and methylene viologen, did not seem to be accepted or reduced by the AAO; and conversely, OYE showed no acceptance of the electron mediators reduced by *Sh*AAO

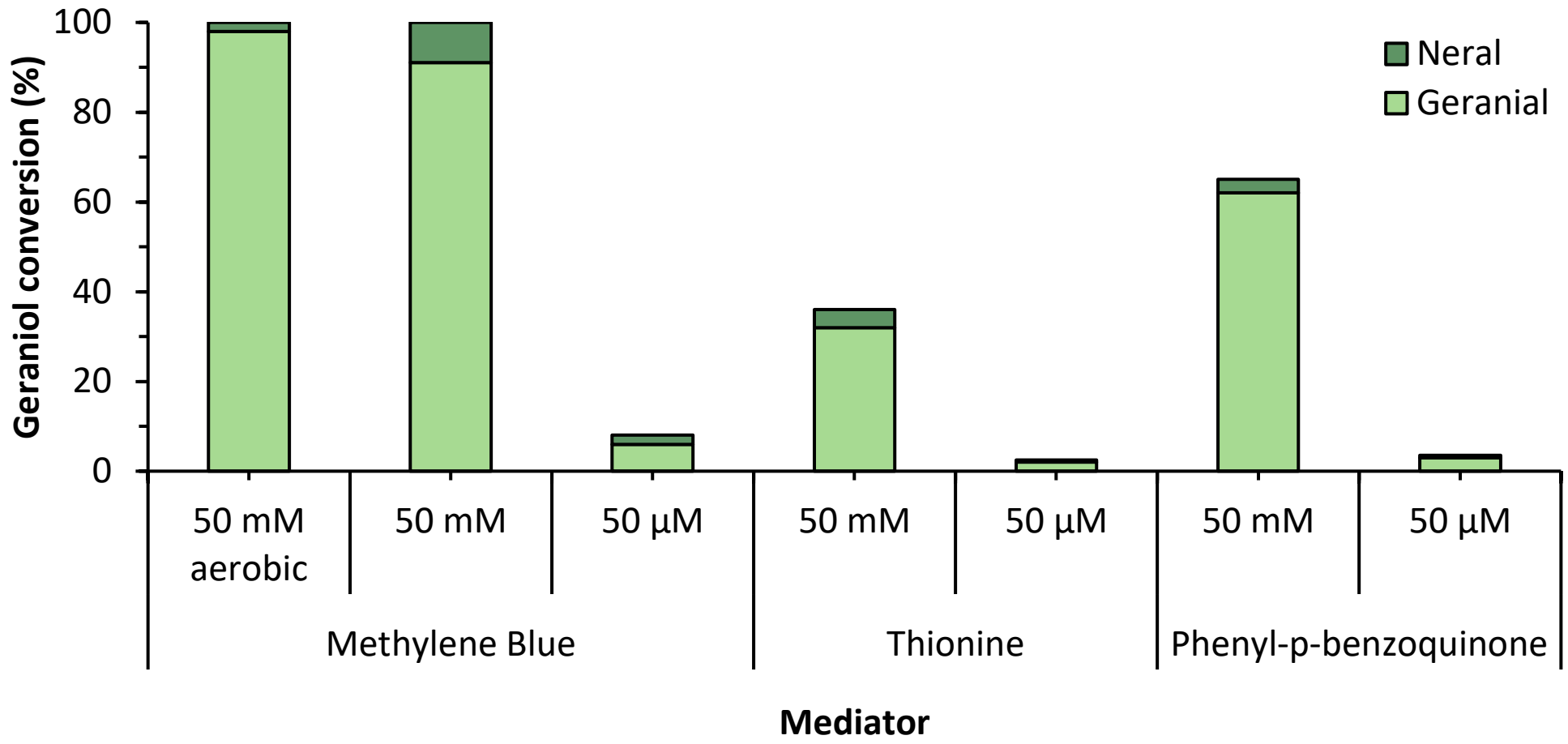

Figure S7. Coupling of both enzymatic systems in a neutral redox system approach.
Conditions: in 1 mL, 50 mM KP*i* pH 8.0, 0.05-50 mM electron mediator, 2.2 µM *Sh*AAO, 5 µM OYE2 Y83V, 10 mM geraniol, at 30 °C and 900 rpm for 4 h under anaerobic atmosphere in a Coy chamber.

GC analyses

GC analyses were carried out on a GC-2010 apparatus (Shimadzu, Japan) equipped with an AOC-20i auto injector and a flame ionisation detector (FID). Products were confirmed by reference standards (geraniol, geranial, neral, (*R*)-citronellal, (*S*)-citronellal, (*R*)- citronellol, (*S*)-citronellol) and analysed using the same column and method (Table S3).

Method 1 column and parameters: Hydrodex ß-TBDAc (Macherey-Nagel, Germany), 50 m × 0.25 mm × 0.25 µm (length, internal diameter, film thickness), injection at 250 °C, split ratio 100, linear velocity 38 cm/sec, column flow 2.3 mL/min, helium as carrier gas.

Method 2 column and parameters: CP-Sil 8 CB, 25 m × 0.25 mm × 1.20 µm, injection at 250 °C, split ratio 50, linear velocity 30 cm/sec, column flow 0.97 mL/min, nitrogen as carrier gas.

Table S3. GC-FID oven program and retention times of identified compounds.

GC method	GC column	Column oven temperatures	Compound	*Rt* (min)
1	CP-Sil 8 CB	120 °C for 3 min<br>5 °C/min to 140 °C hold for 3 min<br>5 °C/min to 165 °C hold for 10 min<br>30 °C/min to 345 °C hold for 1 min	dodecane<br>neral<br>geraniol<br>geranial	13.8<br>15.5<br>15.8<br>16.5
2	Hydrodex ß-TBDAc	60 °C for 5 min<br>5 °C/min to 70 °C hold for 5 min<br>5 °C/min to 80 °C hold for 5 min<br>5 °C/min to 100 °C hold for 5 min<br>5 °C/min to 110 °C hold for 5 min<br>5 °C/min to 120 °C hold for 5 min<br>5 °C/min to 130 °C hold for 5 min<br>5 °C/min to 150 °C hold for 5 min<br>20 °C/min to 230 °C hold for 1 min	dodecane<br>(*S*)-citronellal<br>(*R*)-citronellal<br>(*S*)-citronellol<br>(*R*)-citronellol<br>geraniol<br>neral<br>geranial	29.0<br>42.1<br>42.5<br>46.0<br>46.3<br>49.5<br>52.8<br>57.6
3	Hydrodex ß-TBDAc	60 °C for 5 min<br>5 °C/min to 70 °C hold for 5 min<br>5 °C/min to 80 °C hold for 5 min<br>5 °C/min to 100 °C hold for 5 min<br>5 °C/min to 110 °C hold for 5 min<br>5 °C/min to 120 °C hold for 5 min<br>5 °C/min to 130 °C hold for 5 min<br>5 °C/min to 150 °C hold for 5 min<br>20 °C/min to 230 °C hold for 1 min	Dodecane<br>(*S*)-citronellal<br>(*R*)-citronellal<br>(*S*)-citronellol<br>(*R*)-citronellol<br>geraniol<br>neral<br>geranial	23.0<br>33.4<br>33.8<br>37.1<br>37.3<br>39.9<br>42.8<br>46.6

GC chromatograms

GC Method 1

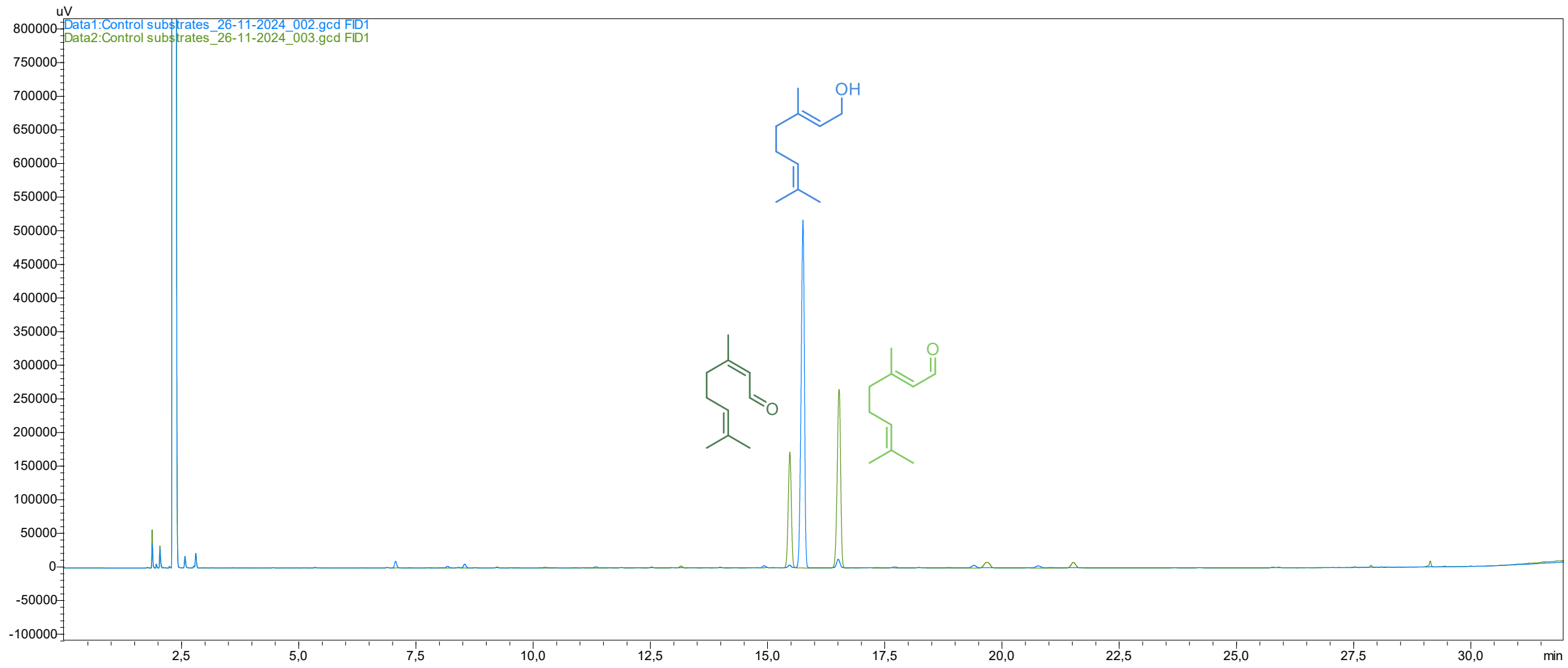

Figure S8. GC chromatograms of standards (Method 1). Dark green: neral, *Rt:* 15.5 min; blue: geraniol, *Rt*: 15.8; and light green: geranial, *Rt:* 16.5 min.

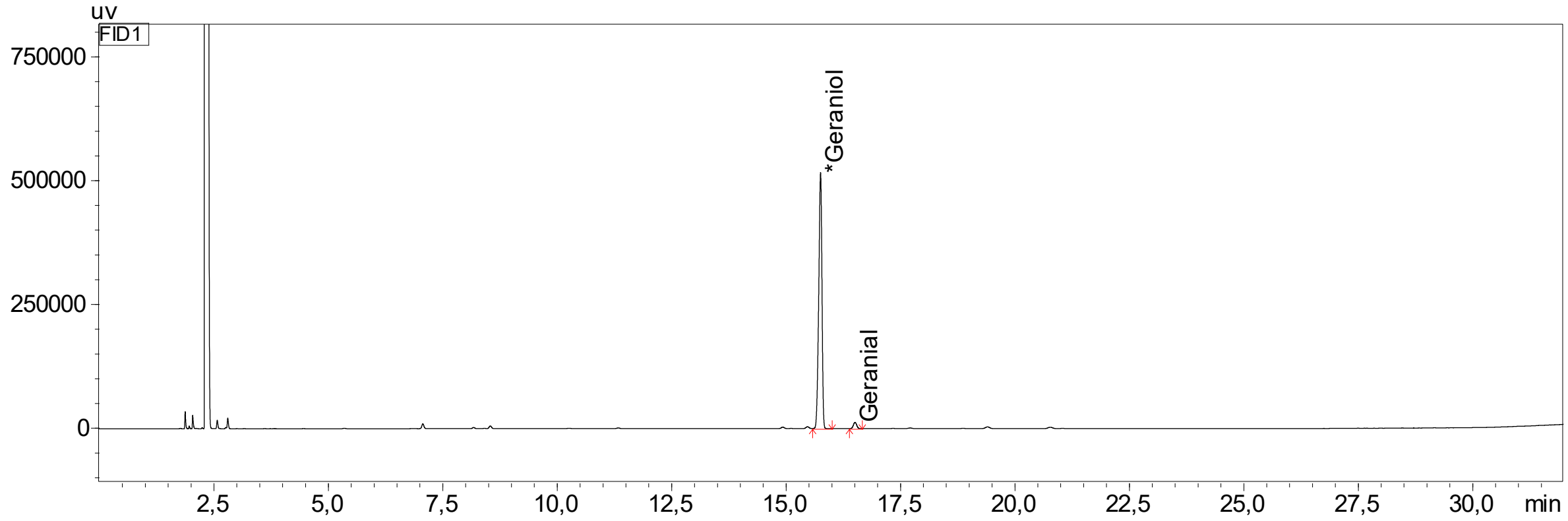

Figure S9. GC chromatogram of the geraniol standard 98% purity (Method 1).

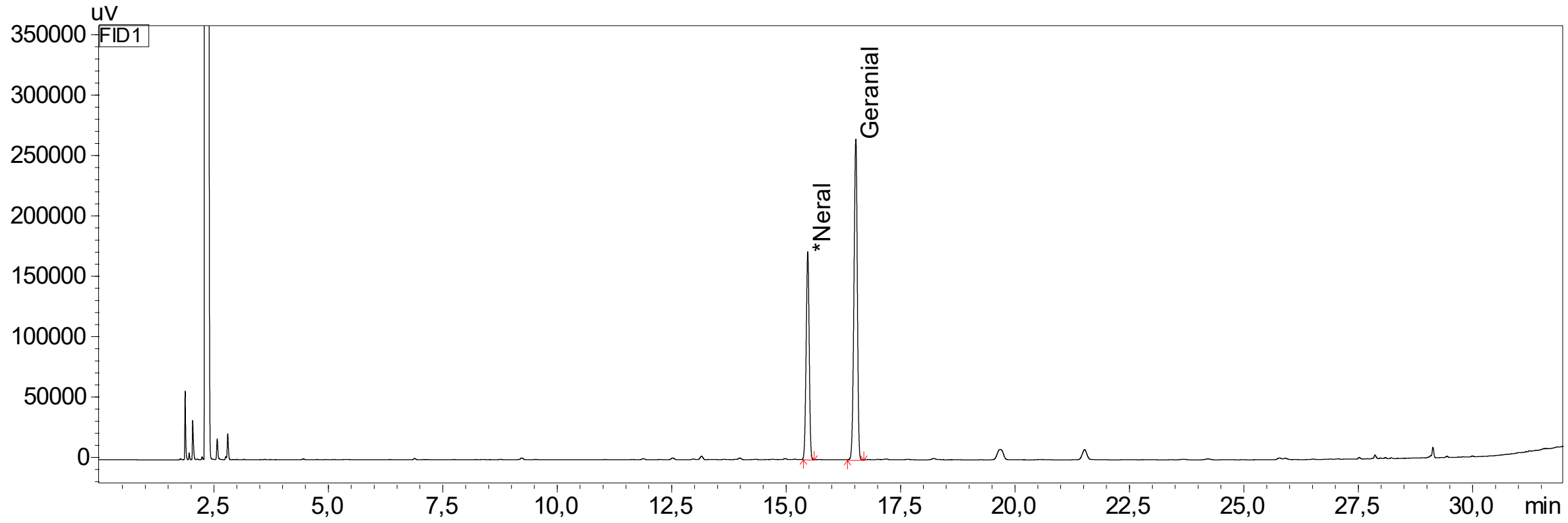

Figure S10. GC chromatogram of citral mix standard (Method 1).

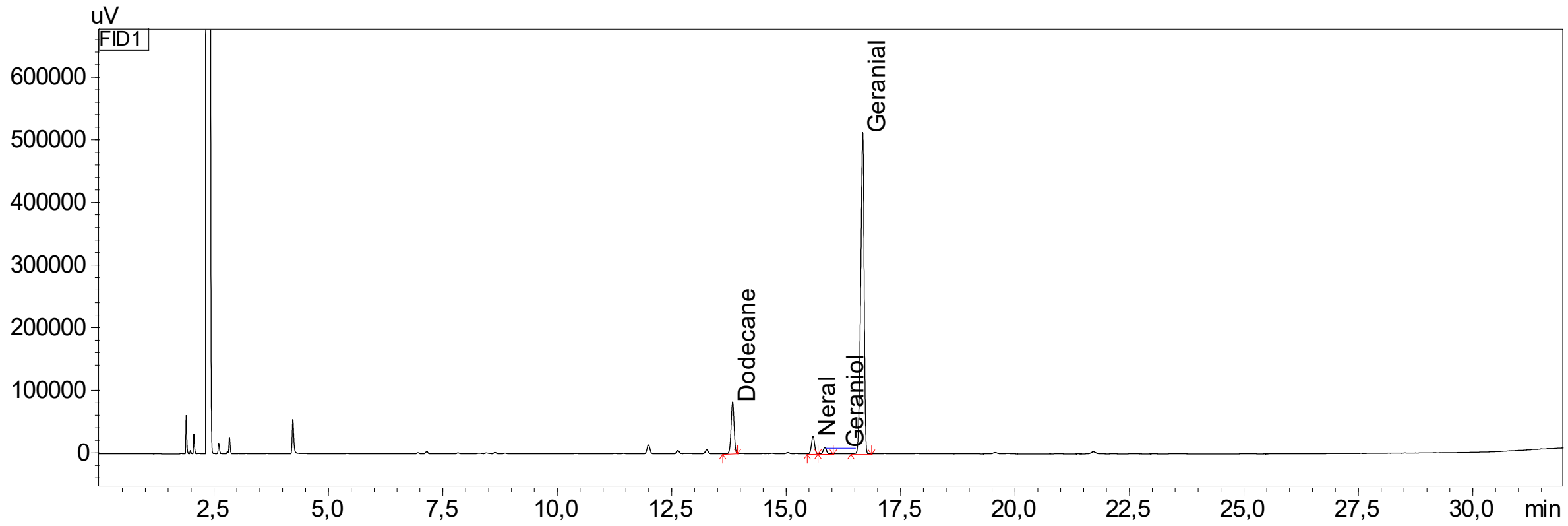

Figure S11. Geraniol oxidation to geranial by *Sh*AAO (Method 1).
Conditions: 50 mM KP*i* pH 8.0, 80 mM geraniol, 8% v/v DMSO, 1000-2500 U/mL catalase, 4.4 µM *Sh*AAO. 1 mL, 30 °C, 800 rpm in a 20 mL glass vial for 18 h.

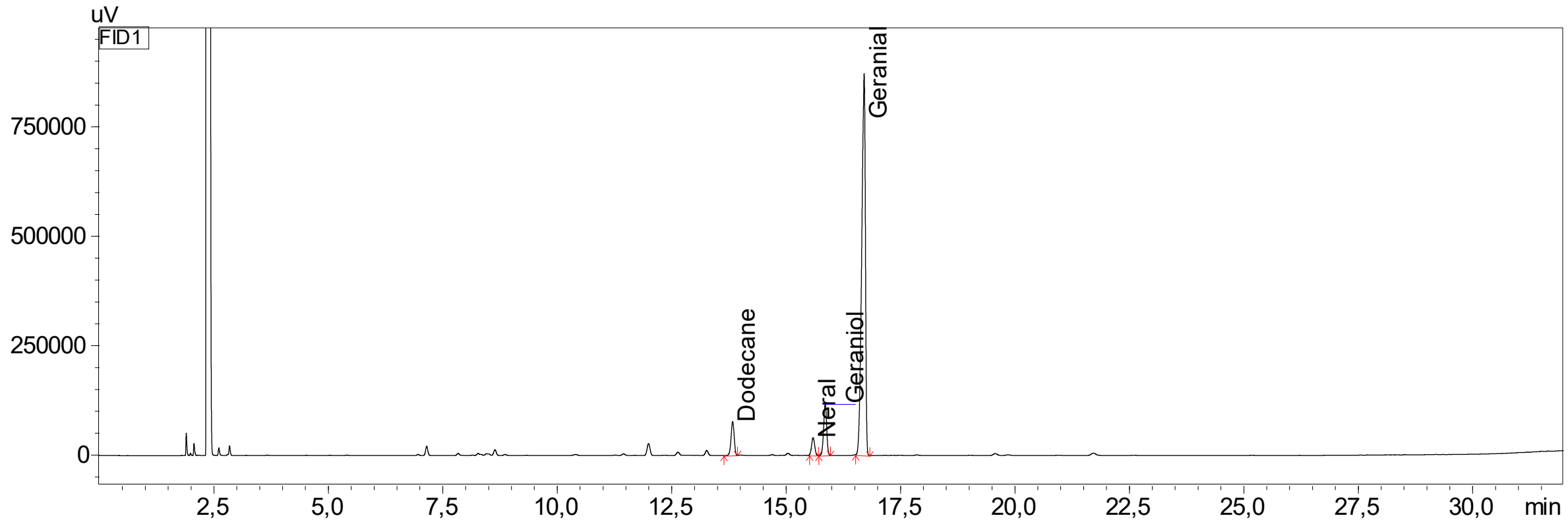

Figure S12. Geraniol oxidation to geranial by *Sh*AAO (Method 1).
Conditions: 50 mM KP*i* pH 8.0, 150 mM geraniol, 1000-2500 U/mL catalase, 4.4 µM *Sh*AAO. 1 mL, 30 °C, 800 rpm in a 20 mL glass vial for 24 h.

GC Method 2

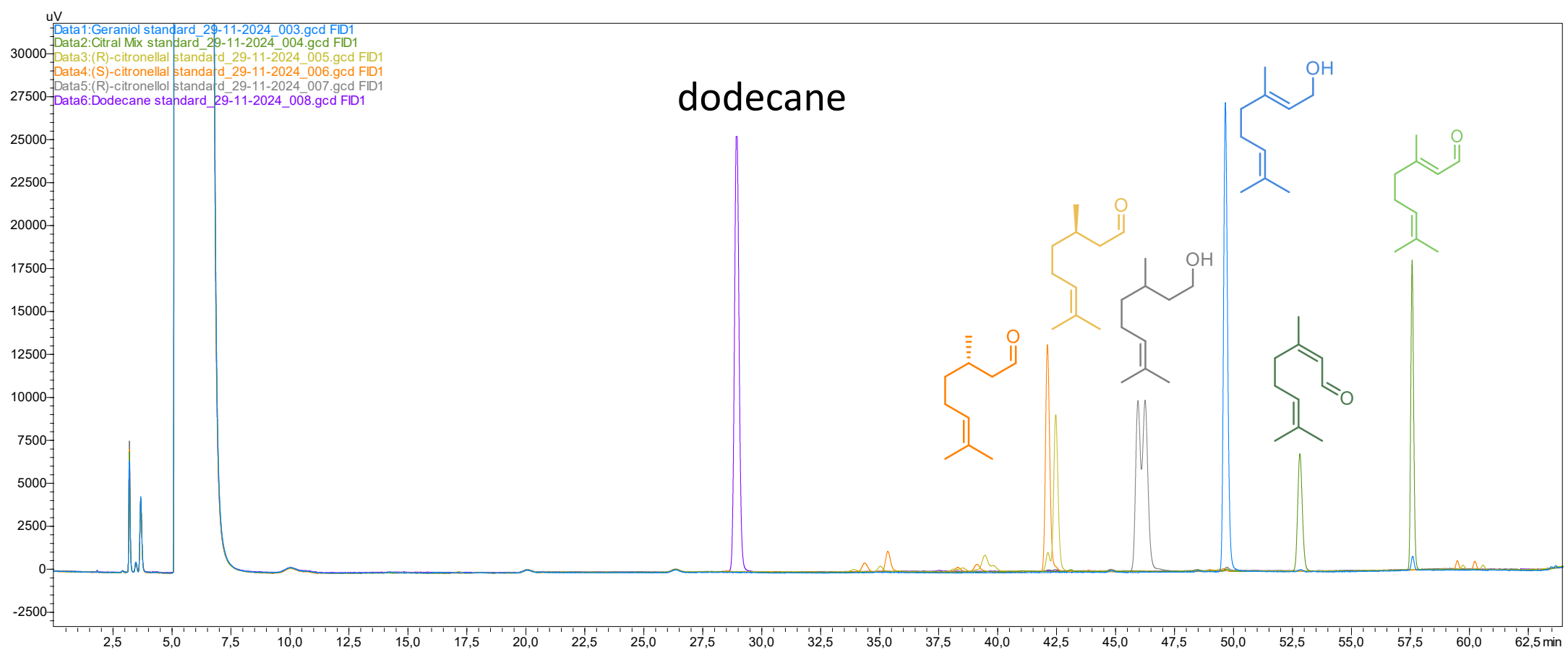

Figure S13. GC chromatograms of standards (Method 2). Purple: dodecane, *Rt*: 29.0 min; orange: (*S*)-citronellal, *Rt:* 42.1 min; yellow, (*R*)-citronellal, *Rt:* 42.5 min; grey: (*S*)-citronellal and (*R*)-citronellol, *Rt:* 46.0 and 46.3 min; dark green: neral, *Rt:* 52.8 min; light green: geranial, *Rt:* 57.6 min.

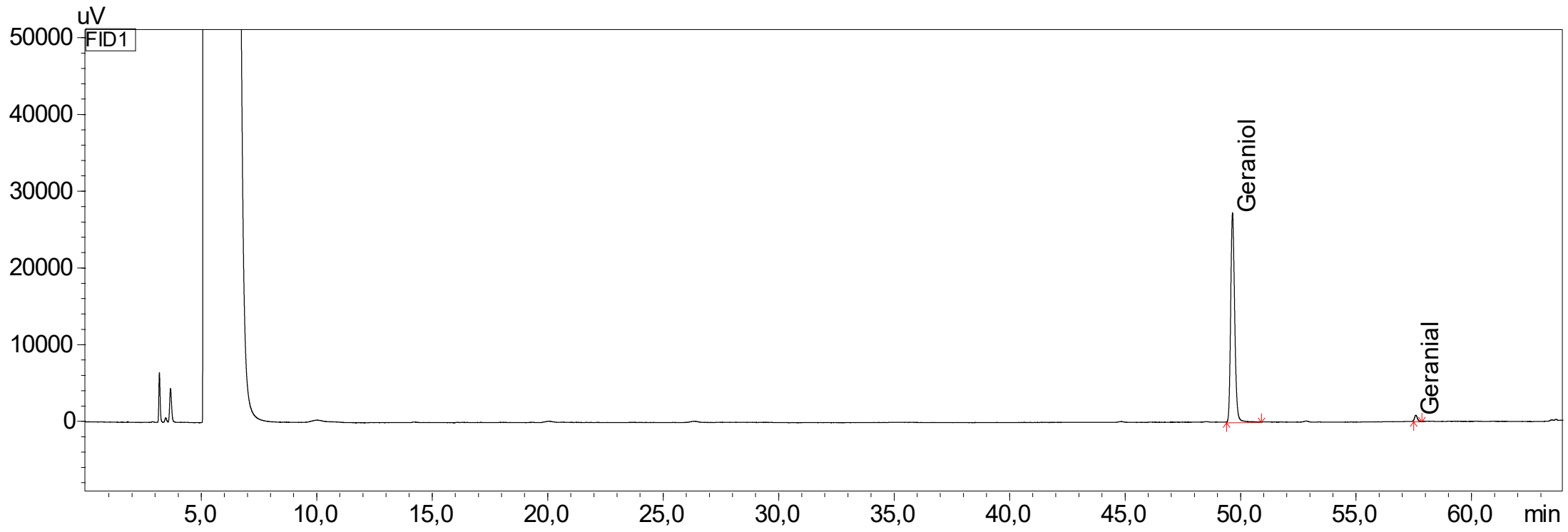

Figure S14. GC chromatogram of the geraniol standard 98% purity (Method 2).

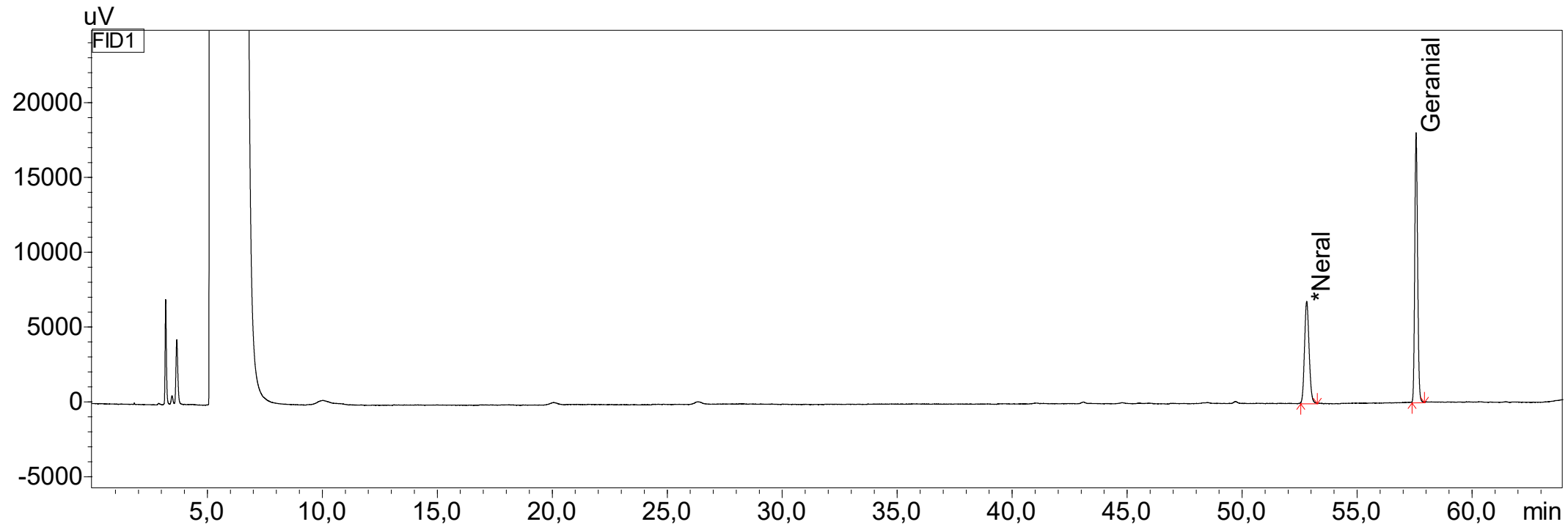

Figure S15. GC chromatogram of citral mix standard (Method 2).

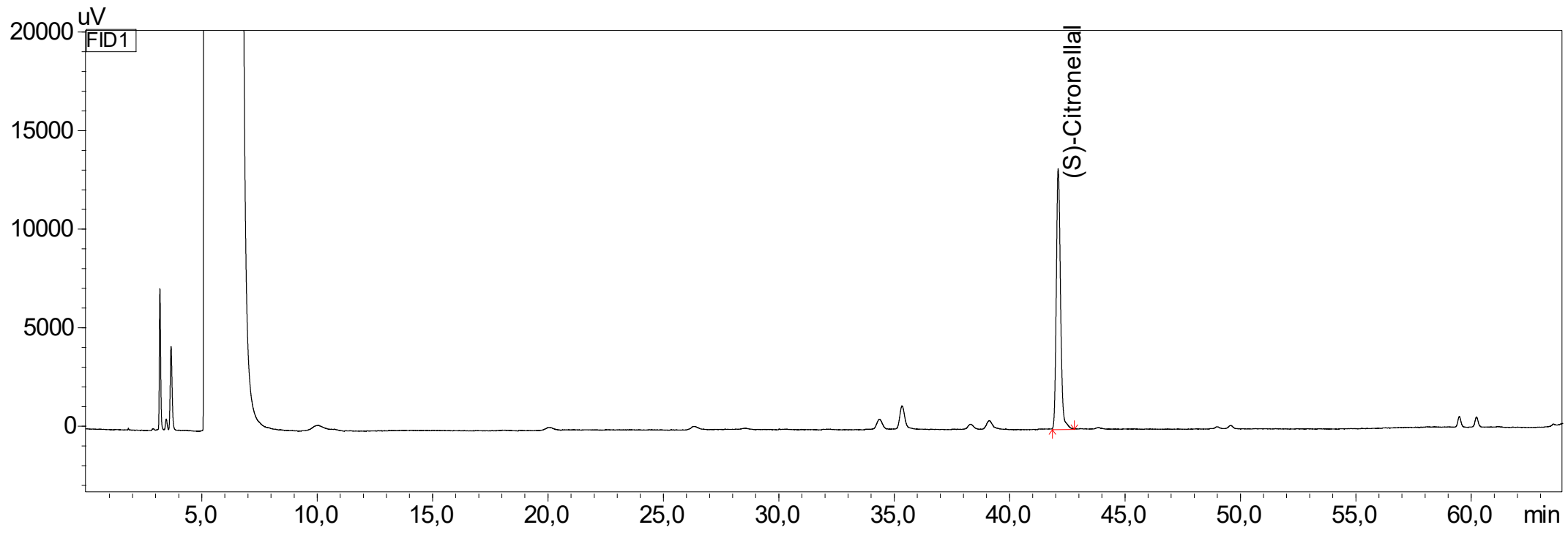

Figure S16. GC chromatogram of (*S*)-citronellal standard (Method 2).

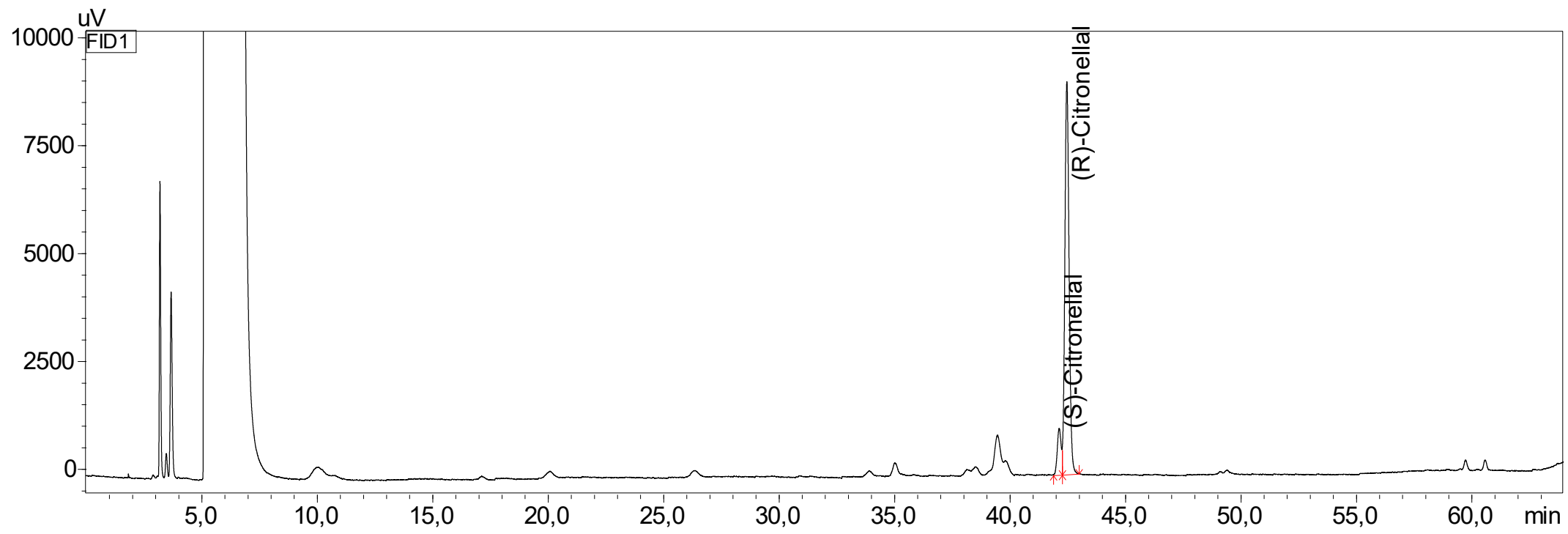

Figure S17. GC chromatogram of (*R*)-citronellal standard (Method 2).

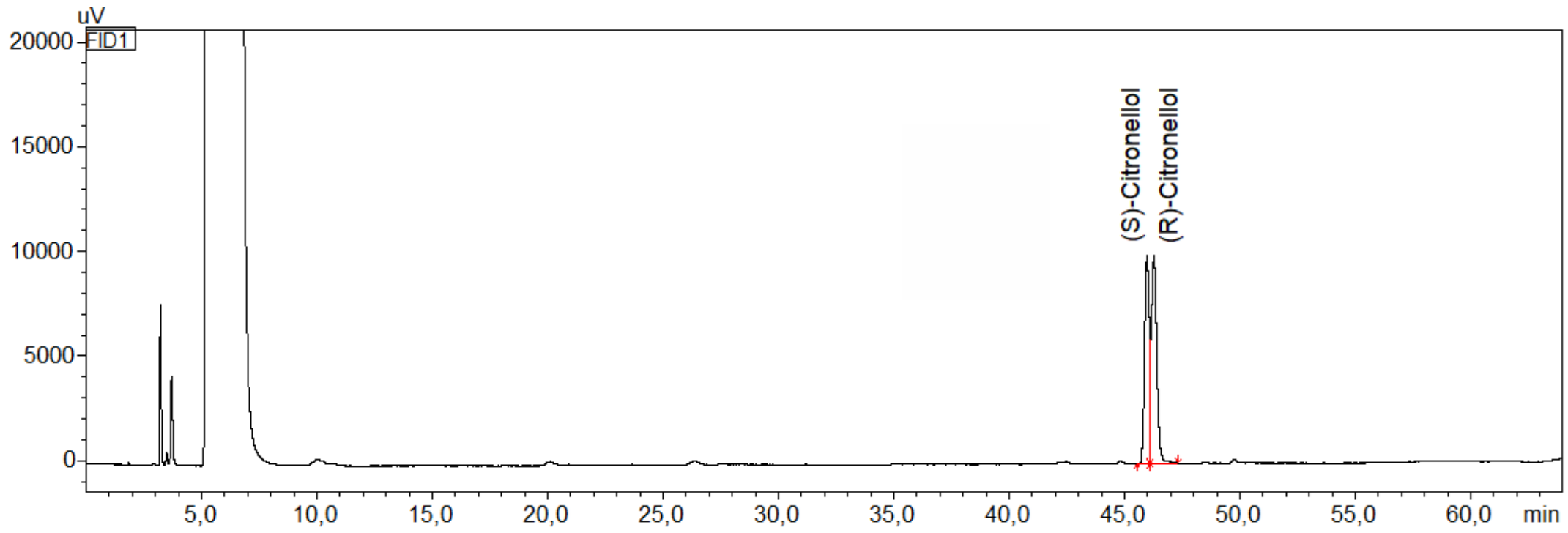

Figure S18. GC chromatogram of (*S*)- and (*R*)-citronellol (Method 2).

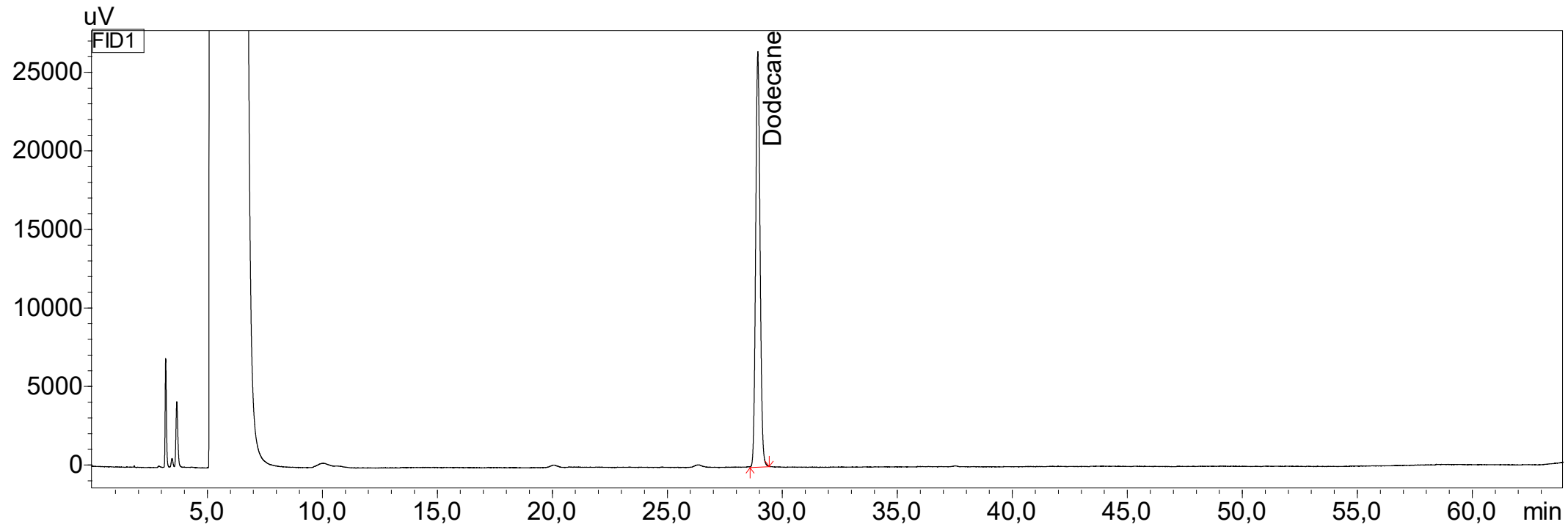

Figure S19. GC chromatogram of dodecane, internal standard (Method 2).

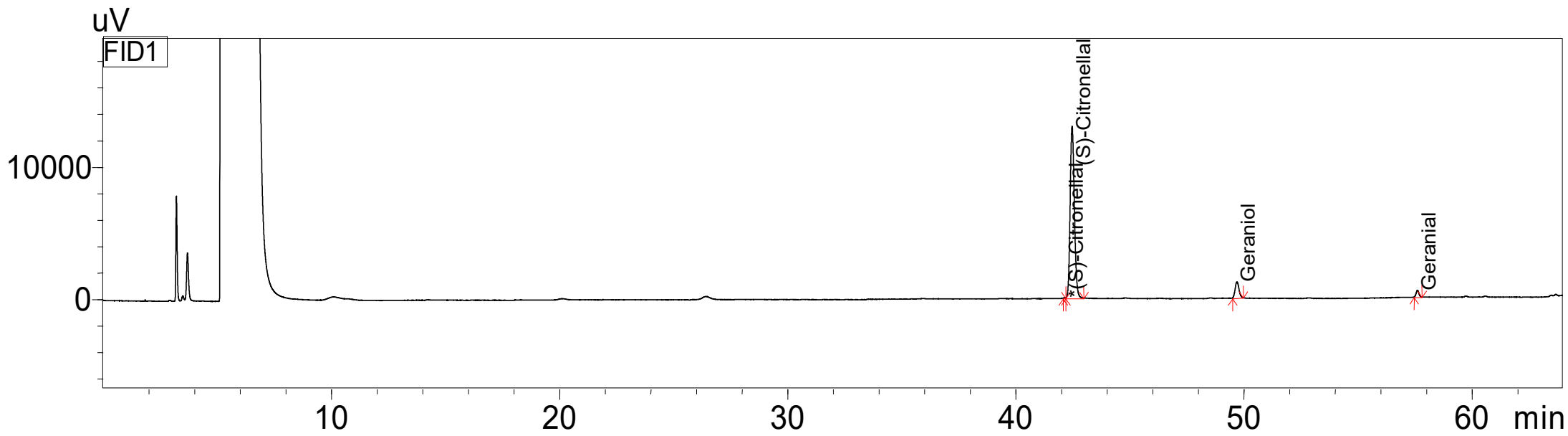

Figure S20. GC chromatogram of the full cascade (Method 2).
Conditions: In 1 mL, 50 mM KP*i* pH 8.0, 100 mM geraniol, 1000-2500 U/mL catalase, 2.2 µM *Sh*AAO, 10 µM OYE2 Y83V, 0.2 mM NAD$^+$, 10 U/mL *Bs*GDH, 200 mM Glc in a 20 mL glass vial at 30 °C and 900 rpm for 24 h.

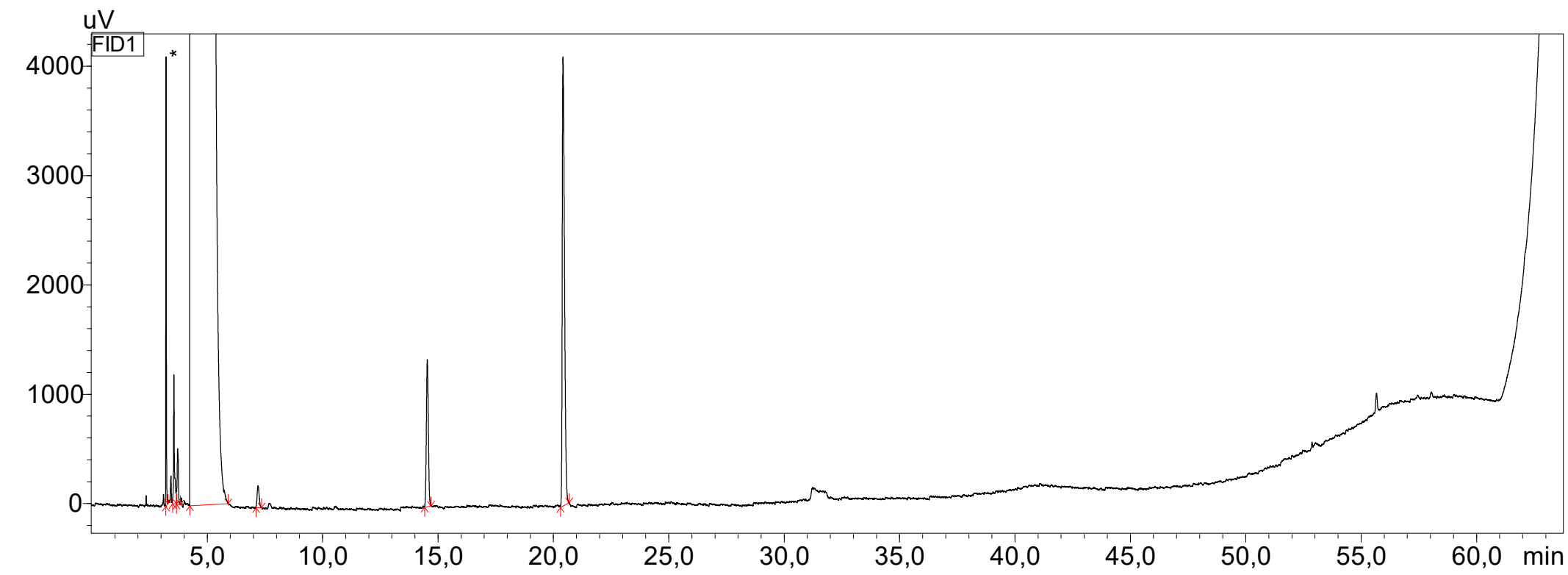

Figure S21. GC chromatogram of EtOAc solvent, showing background extra peaks present on the column (Method 2).

GC Method 3

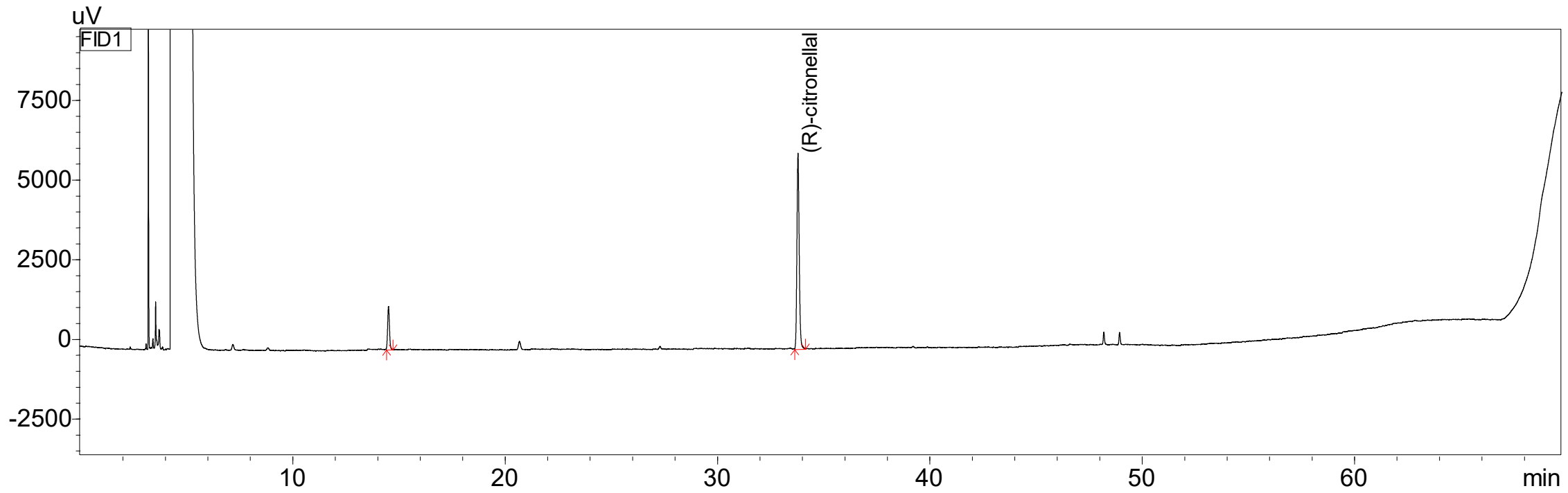

Figure S22. GC chromatogram of the full cascade product after column chromatography purification, 98% purity (Method 3). Conditions: In 1 mL, 50 mM KP*i* pH 8.0, 100 mM geraniol, 1000-2500 U/mL catalase, 2.2 µM *Sh*AAO, 10 µM OYE2 Y83V, 0.2 mM NAD^+, 10 U/mL *Bs*GDH, 200 mM Glc in a 20 mL glass vial at 30 °C and 900 rpm for 24 h.

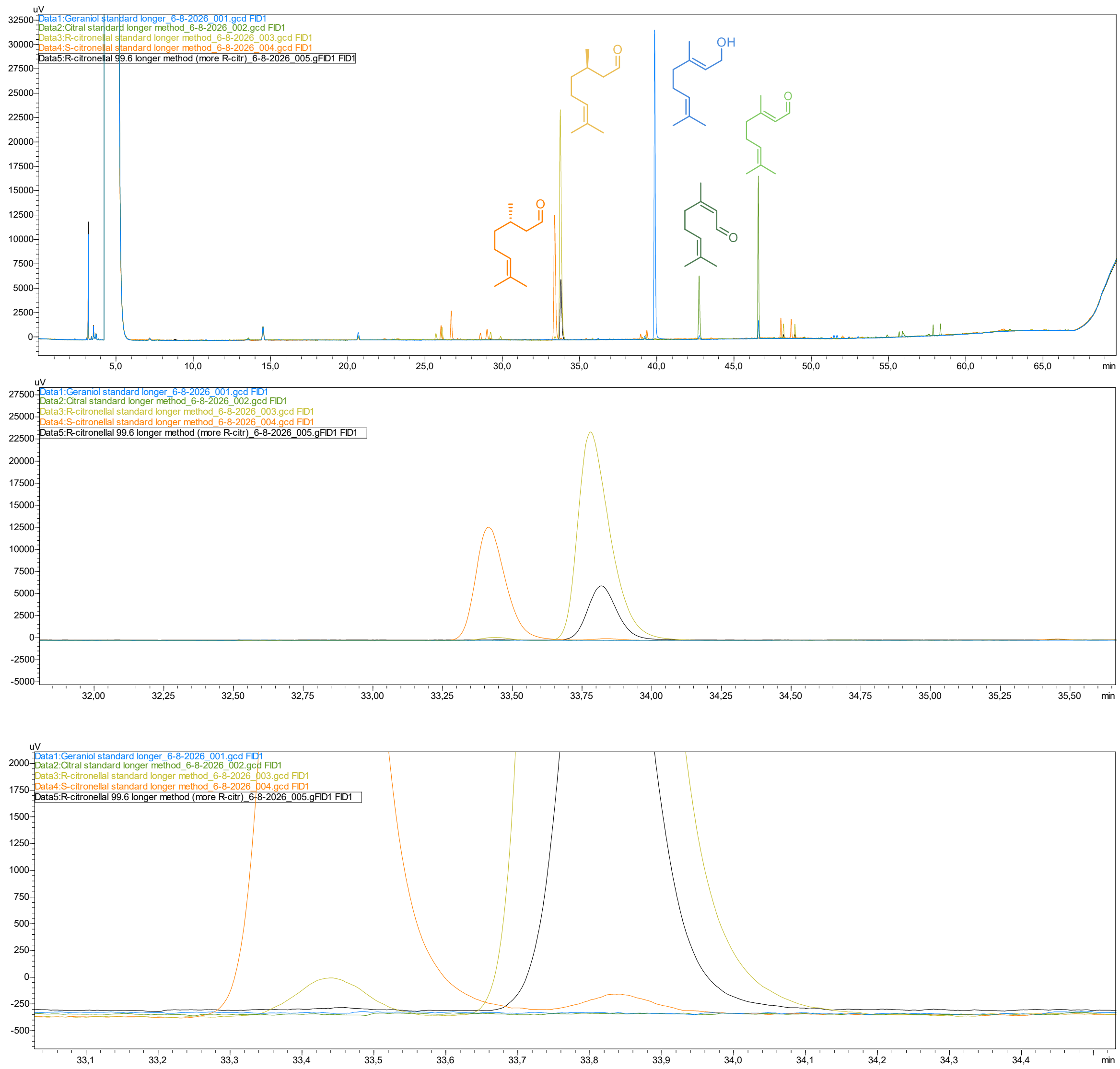

Figure S23. GC chromatograms of standards and column-purified (*R*)-citronellal from the chromatogram of **Figure S22** (Method 3). Orange: (*S*)-citronellal, *Rt:* 33.4 min; yellow: (*R*)-citronellal, *Rt:* 33.8 min; blue: geraniol, *Rt*: 39.9 min; dark green: neral, *Rt:* 42.8 min; light green: geranial, *Rt:* 46.6 min. Top: full chromatogram. Middle and Bottom: Zoomed chromatogram.

Calibration curves

Below can be observed the calibration curves used during this research for Method 1 (**Figure S24**) and Method 2 (**Figure S25**)

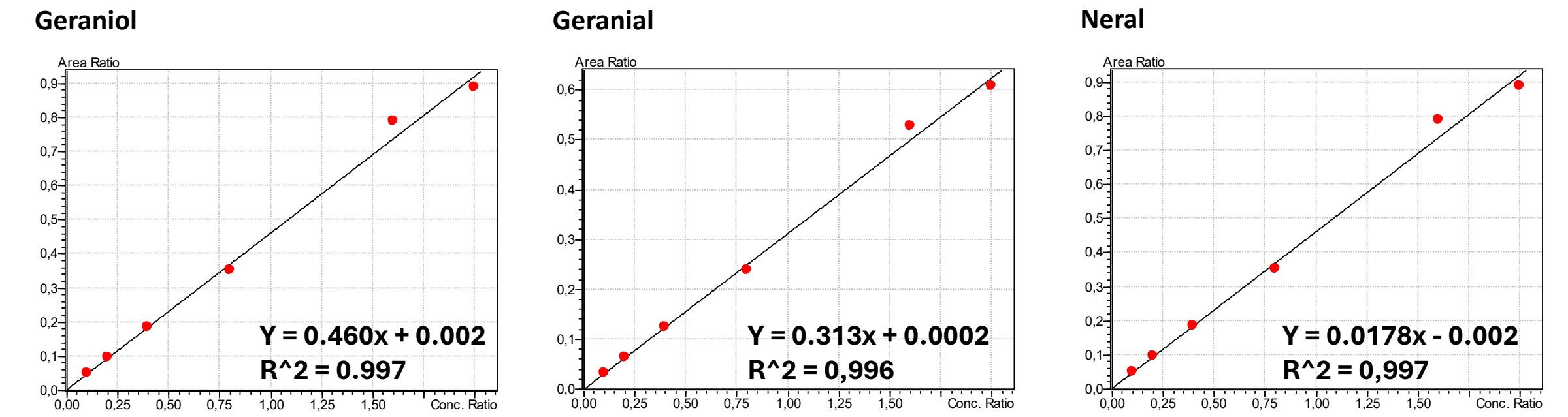

Figure S24. Calibration curve for GC Method 1.

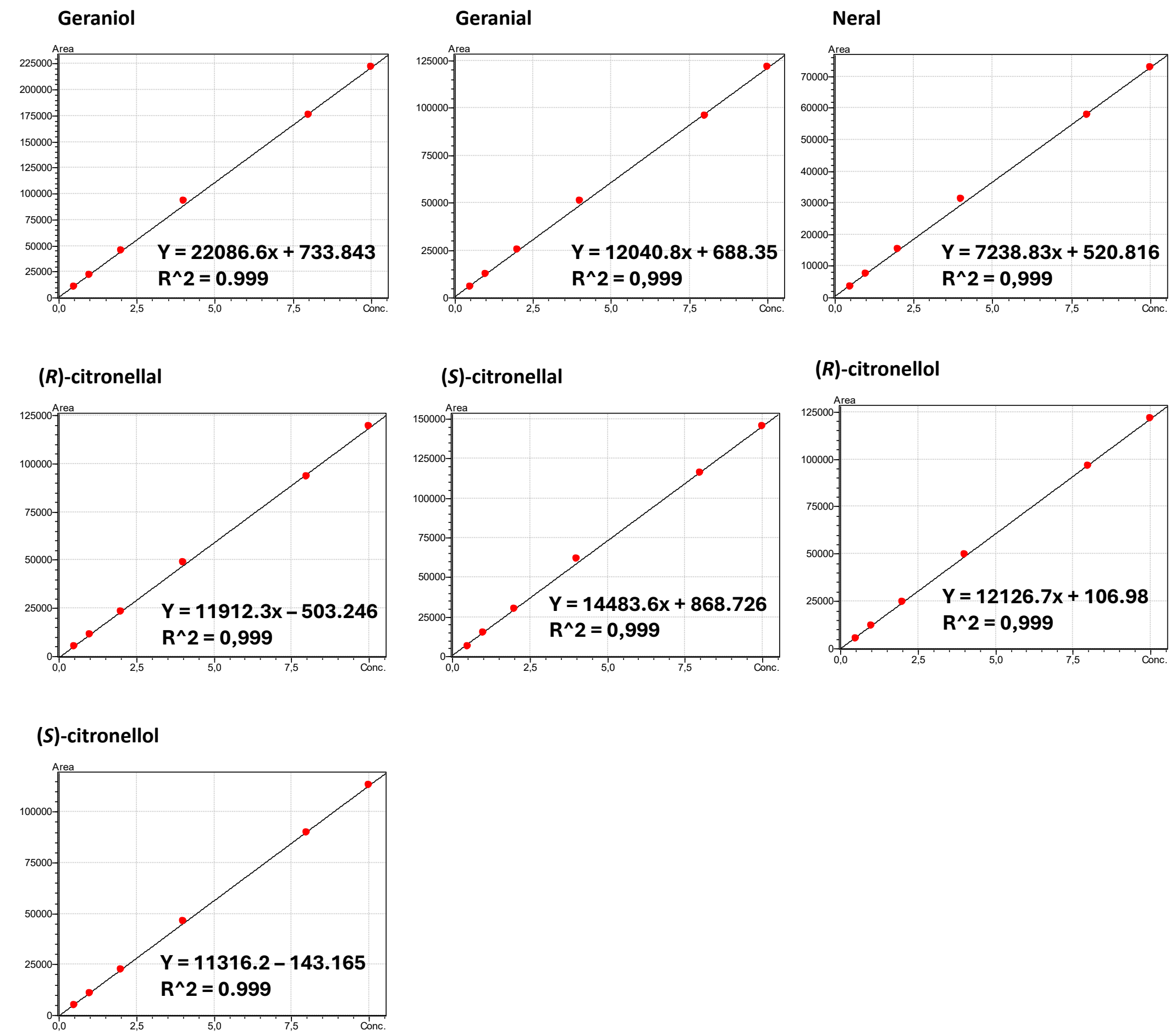

Figure S25. Calibration curve for GC Method 2.

NMR spectra

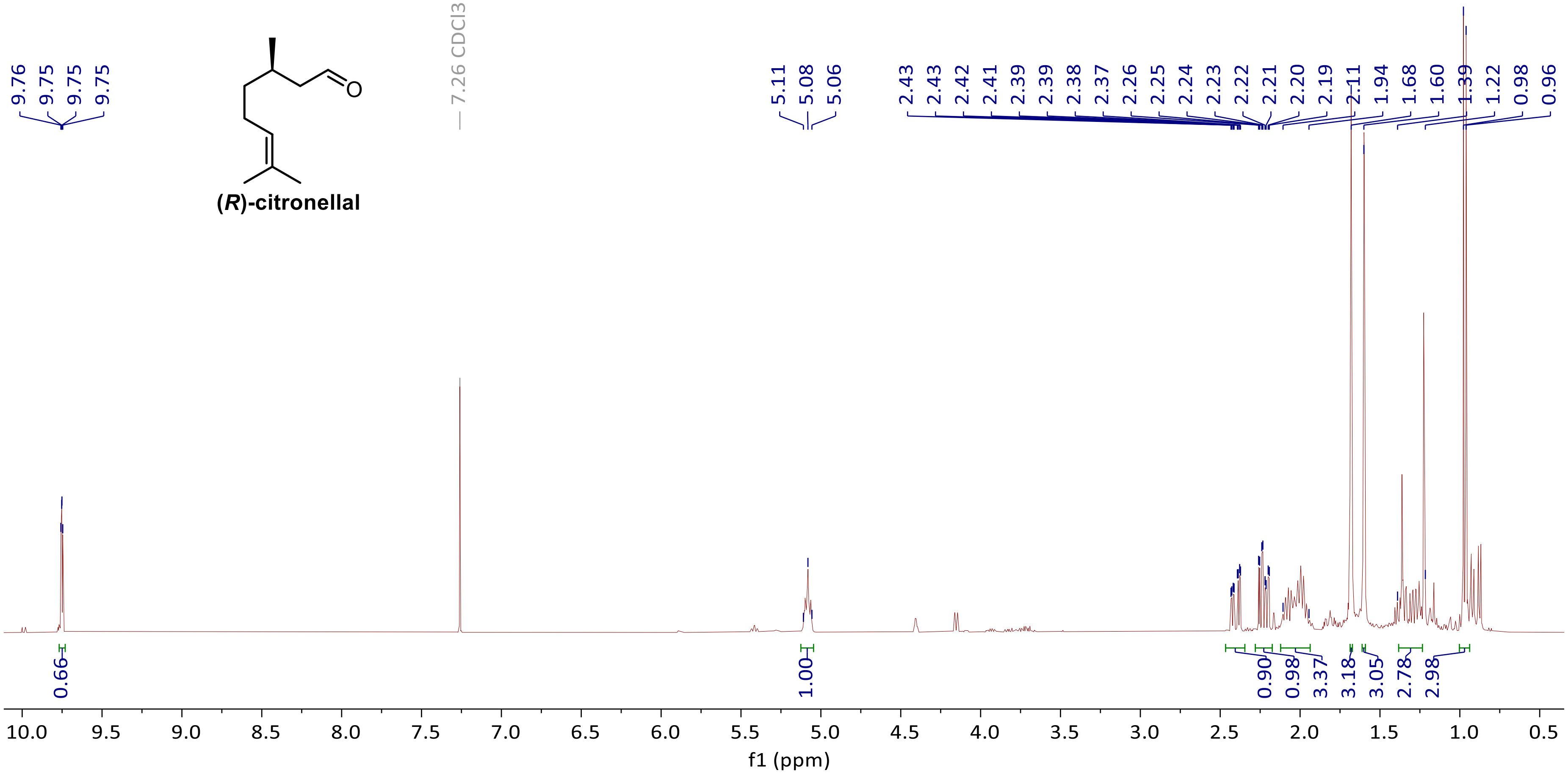

Figure S26. ^1H NMR spectrum of isolated (*R*)-citronellal from the scale-up reaction. 98% purity according to GC (**Figure S22**).

^1H NMR (400 MHz, $CDCl_3$) δ 9.75 (dd, *J* = 2.7, 2.0 Hz, 1H, CHO), 5.11-5.06 (m, 1H, CH=C), 2.43-2.37 (ddd, *J* = 16.0, 5.6, 2.1 Hz, 1H), 2.26-2.19 (ddd, *J* = 16.0, 8.0, 2.7 Hz, 1H), 2.10-1.94 (m, 2H), 1.68 (s, 3H), 1.60 (s, 3H), 1.39-1.22 (m, 2H), 0.98 (d, *J* = 6.7 Hz, 3H).

References

(1) Ferrer-Carbonell, C.; Villa, R.; Viskaal, I.; Opperman, D. J.; Paul, C. E. Enzymatic synthesis of enantiopure (*R*)-citronellal from geraniol via a short-chain dehydrogenase and ene reductase. *Adv. Synth. Catal.* **2025**, *367*, e202500060.

(2) Wolder, A. E.; Heckmann, C. M.; Hagedoorn, P.-L.; Opperman, D. J.; Paul, C. E. Asymmetric monoreduction of α,β-dicarbonyls to α-hydroxy carbonyls by ene reductases. *ACS Catal.* **2024**, *14*, 15713–15720.

(3) Ascaso-Alegre, C.; Cinca-Fernando, P.; Roberts, T. L.; López-Fernández, P.; Herrera, R. P.; Cosgrove, S. C.; Ferreira, P.; Mangas-Sánchez, J. Scope and synthetic applications of the aryl-alcohol oxidase from *Streptomyces hiroshimensis* (*Sh*AAO). *Org. Lett.* **2025**, *27*, 12086–12091.

(4) Paul, C. E.; Tischler, D.; Riedel, A.; Heine, T.; Itoh, N.; Hollmann, F. Nonenzymatic regeneration of styrene monooxygenase for catalysis. *ACS Catal.* **2015**, *5*, 2961–2965.

(5) Cinca-Fernando, P.; Ascaso-Alegre, C.; Sevilla, E.; Martínez-Júlvez, M.; Mangas-Sánchez, J.; Ferreira, P. Discovery, characterization, and synthetic potential of two novel bacterial aryl-alcohol oxidases. *Appl. Microbiol. Biotechnol.* **2024**, *108*, 498.

(6) Rauch, M. C. R.; Gallou, Y.; Delorme, L.; Paul, C. E.; Arends, I. W. C. E.; Hollmann, F. Metals in biotechnology: Cr-driven stereoselective reduction of conjugated C=C double bonds. *ChemBioChem* **2020**, *21*, 1112–1115.

(7) Joseph Srinivasan, S.; Cleary, S. E.; Ramirez, M. A.; Reeve, H. A.; Paul, C. E.; Vincent, K. A. *E. coli* nickel-iron hydrogenase 1 catalyses non-native reduction of flavins: demonstration for alkene hydrogenation by Old Yellow Enzyme ene-reductases. *Angew. Chem. Int. Ed.* **2021**, *60*, 13824–13828.

(8) Al-Shameri, A.; Willot, S. J.-P.; Paul, C. E.; Hollmann, F.; Lauterbach, L. H_2 as a fuel for flavin- and H_2O_2-dependent biocatalytic reactions. *Chem. Commun.* **2020**, *56*, 9667–9670.

(9) Efimov, I.; Cronin, C. N.; McIntire, W. S. Effects of noncovalent and covalent FAD binding on the redox and catalytic properties of p-cresol methylhydroxylase. *Biochemistry* **2001**, *40*, 2155–2166.

(10) Mao, H.-B.; Li, G.-F.; Ruan, X.; Wu, Q.-Y.; Gong, Y.-D.; Zhang, X.-F.; Zhao, N.-M. The redox state of plastoquinone pool regulates state transitions via cytochrome b_6f complex in *Synechocystis* sp. PCC 6803. *FEBS Lett.* **2002**, *519*, 82–86.
